# Transcriptional variability accelerates preleukemia by cell diversification and perturbation of protein synthesis

**DOI:** 10.1126/sciadv.abn4886

**Published:** 2022-08-03

**Authors:** Shikha Gupta, Oliver M. Dovey, Ana Filipa Domingues, Oliwia W. Cyran, Caitlin M. Cash, George Giotopoulos, Justyna Rak, Jonathan Cooper, Malgorzata Gozdecka, Liza Dijkhuis, Ryan J. Asby, Noor Al-Jabery, Victor Hernandez-Hernandez, Sudhakaran Prabakaran, Brian J. Huntly, George S. Vassiliou, Cristina Pina

**Affiliations:** ^1^ Department of Genetics, University of Cambridge, Cambridge, UK.; ^2^ Department of Haematology, University of Cambridge, Cambridge, UK.; ^3^ Wellcome Sanger Institute, Wellcome Trust Genome Campus, Cambridge, UK.; ^4^ Wellcome Trust-MRC Cambridge Stem Cell Institute, University of Cambridge, Cambridge, UK.; ^5^ College of Health, Medicine and Life Sciences - Division of Biosciences, Brunel University London, Uxbridge, UK.; ^6^ Centre for Genome Engineering and Maintenance, Brunel University London, Uxbridge, UB8 3PH, UK.; ^7^ NonExomics Inc., Boston, MA, USA.

## Abstract

Transcriptional variability facilitates stochastic cell diversification and can in turn underpin adaptation to stress or injury. We hypothesize that it may analogously facilitate progression of premalignancy to cancer. To investigate this, we initiated preleukemia in mouse cells with enhanced transcriptional variability due to conditional disruption of the histone lysine acetyltransferase gene *Kat2a*. By combining single-cell RNA sequencing of preleukemia with functional analysis of transformation, we show that *Kat2a* loss results in global variegation of cell identity and accumulation of preleukemic cells. Leukemia progression is subsequently facilitated by destabilization of ribosome biogenesis and protein synthesis, which confer a transient transformation advantage. The contribution of transcriptional variability to early cancer evolution reflects a generic role in promoting cell fate transitions, which, in the case of well-adapted malignancies, contrastingly differentiates and depletes cancer stem cells. That is, transcriptional variability confers forward momentum to cell fate systems, with differential multistage impact throughout cancer evolution.

## Teaser

Loss of Kat2a enhances transcriptional variability of ribosome biosynthetic programs and transiently accelerates preleukemia.

## INTRODUCTION

Tumors evolve by genetic drift and natural selection (*1*, *2*). Acquisition of new mutations confers a probability of adaptation to new environmental pressures (*3*), facilitates progression and transformation of premalignant lesions, promotes metastasis, and drives treatment resistance (*4*). In recent years, it became apparent that nongenetic instability, in particular variability in methylation epialleles, can confer adaptive advantages to tumor growth and survival irrespective of mutations and function as driver of therapy resistance and disease relapse in hematological malignancies (*5*, *6*). Hematological malignancies, and, in particular, acute myeloid leukemia (AML), are strongly dependent on epigenetic regulation, both through mutation of chromatin factors and by co-option of unmutated chromatin regulators into maintenance of leukemogenic programs (*7*–*9*). Notably, AML has lower levels of mutations than solid tumors, supporting the notion that nongenetic events may be especially important in the former (*7*). Akin to genetic instability, epigenetic variability is increased in leukemia initiation and relapse but low in leukemia maintenance (*10*, *11*), suggesting that reconfiguration of molecular/transcriptional programs may perturb the identity or survival of well-adapted leukemia cells by disrupting pro-oncogenic molecular signatures. We have recently captured this phenomenon upon loss of KAT2A (lysine acetyltransferase 2A), a histone acetyltransferase that promotes gene transcription through activation of transcriptional bursting and stabilization of gene expression levels. *Kat2a* loss (NULL) results in enhanced cell-to-cell transcriptional variability and progressive loss of leukemia stem cells (LSCs) transformed with the *KMT2A-MLLT3* (*MLL-AF9*) gene fusion (*12*). Accordingly, KAT2A is required for maintenance of AML cell lines and in vitro self-renewal of patient AML blasts (*13*). At a cellular level, loss of *Kat2a* results in perturbation of leukemia lineage trajectories, with emergence of multiple incongruent differentiation pathways that deplete LSC but fail to uniformly differentiate leukemia cells (*12*). A similar pattern of incongruous exit from the stem cell state was observed upon KAT2A inhibition in mouse embryonic stem (ES) cells (*14*). MLL-AF9 results in an aggressive leukemia, both in mice and in humans, and requires minimal cooperativity from additional mutational events (*7*, *15*). Hence, it provides a good representation of a well-adapted leukemia, with minimal genetic and epigenetic variability. However, it does not reflect what is observed with more common forms of AML such as those associated with *RUNX1-RUNX1T1* (*AML1-ETO*), where progression in mouse models is slow and infrequent (*7*, *16*), or clonal hematopoiesis, in which the associated mutations (e.g., in *IDH1/2*, *TET2*, *DNMT3A*) convey a self-renewal advantage but require additional genetic events for leukemia (*7*, *16*). In these cases, we postulate that malignant progression may be facilitated by nongenetic instability, which can be promoted through loss of *Kat2a*. We tested this hypothesis through investigation of two preleukemia mouse models *Idh1^R132H^* and *RUNX1-RUNX1T1*[RT1(9a)] (*17*), which together represent up to 25% of human AML disease (*7*, *16*, *18*). We compared the effects of the respective mutations in the presence and absence of *Kat2a* and integrated functional in vitro and in vivo transformation assays with single-cell RNA sequencing (scRNA-seq) analysis, to illuminate consequences on transcriptional variability and differentiation trajectories and explain differential transformation progression.

## RESULTS

### Loss of *Kat2a* facilitates *IDH1^R132H^* preleukemia transformation

First, we developed a new inducible *Idh1^R132H^* allele (fig. S1, A to C) and crossed it into an *Mx1-Cre* background (fig. S1D), to activate the mutation in hematopoietic tissues. We verified the functionality of the *Idh1^R132H^* allele by accumulation of the oncometabolite 2-hydroxyglutarate (fig. S1, E and F). *Idh1*^R132H^ mice develop leukemia rarely, with long latency and low penetrance, with no significant effects on overall survival (fig. S1G). In contrast, combination of *Idh1^R132H^* with other leukemogenic mutations, namely, *NRas* and *Npm1c* (triple-mutant), results in short-latency high-penetrance leukemia development (fig. S1G), confirming the preleukemic nature of the *Idh1^R132H^* model. Accordingly, triple-mutant bone marrow (BM) cells, but not cells with *Idh1^R132H^* alone, have enhanced colony-forming cell (CFC) assay–replating ability, an in vitro measure of transformation (fig. S1H). Comparison of RNA-seq from triple-mutant leukemias versus triple-mutant preleukemias, or versus *Idh1^R132H^* alone, revealed a gene signature that was specific to the leukemia state and in which down-regulated genes were enriched for KAT2A chromatin targets (fig. S1I). This association suggests that loss of KAT2A activity may contribute to progression of preleukemia to overt AML.

**Fig. 1.**
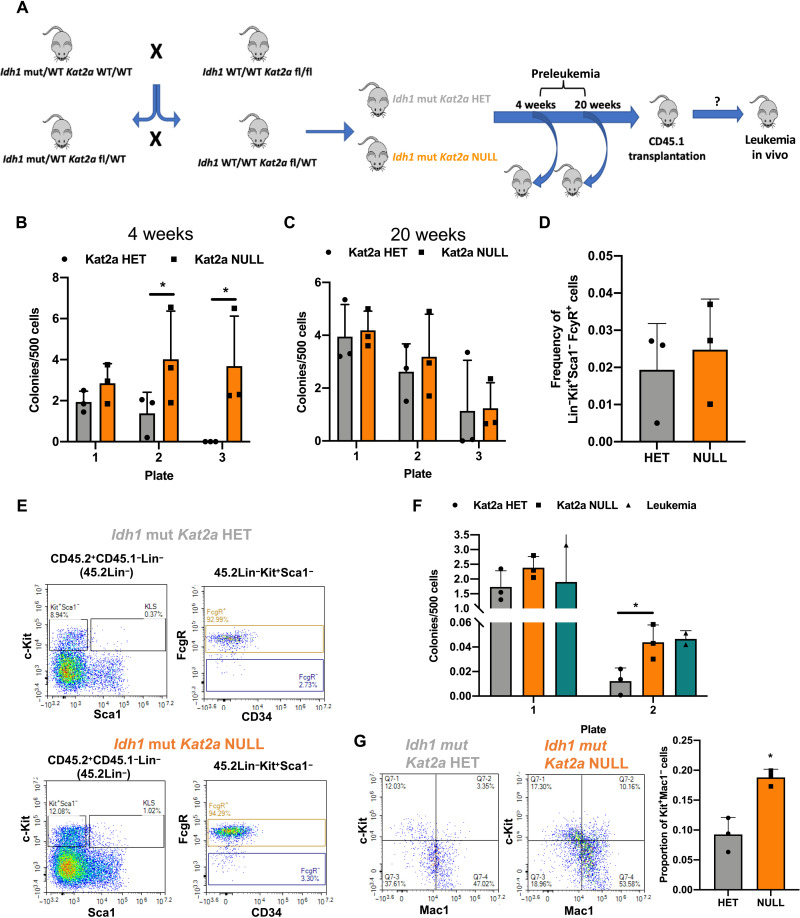
*Kat2a* loss facilitates development of *Idh1^R132H^* preleukemia. (**A**) Diagram of *Idh1^R132H^* (*Idh1* mut) and *Kat2a*^fl/fl^ mouse crosses to generate *Idh1* mut *Kat2a* HET and *Idh1* mut *Kat2a*NULL cells used in preleukemia studies. WT, wild-type. (**B**) CFC assays of *Idh1* mut *Kat2a* HET and NULL BM cells 4 weeks after polyinosinic:polycytidylic acid (pIpC) treatment. Mean ± SD, *n* = 3. (**C**) CFC assays of *Idh1* mut *Kat2a* HET and NULL BM cells 20 weeks after pIpC treatment; mean ± SD, *n* = 3. (**D**) Quantification of GMP-like BM cells obtained from *Idh1* mut CD45.2^+^ grafts; mean ± SD, *n* = 3 irradiated recipients (CD45.1). (**E**) Representative flow cytometry plots of BM cells in (D). Top: *Idh1* mut *Kat2a* HET. Bottom: *Idh1* mut *Kat2a*NULL. KLS, Lin-Kit^+^Sca1^+^. (**F**) Serial replating CFC assays of *Idh1* mut BM grafts. Mean ± SD, *n* = 3 *Idh1* mut *Kat2a* HET and NULL and *n* = 2 *Idh1* mut leukemia. (**G**) Flow cytometry of colonies in (F). Left: representative plots. Right: Kit^+^Mac1^−^ progenitor quantification. Mean ± SD, *n* = 3. All analyses two-tailed *t* test, *P < 0.05.

To investigate this putative contribution of *Kat2a* loss to preleukemia progression, we crossed conditional *Idh1^R132H^* and *Kat2a^Flox/Flox^* mice, into the Mx1-Cre background (Fig. 1A), to generate *Idh1^R132H^* animals that were heterozygous (HET) or NULL for *Kat2a* (fig. S2, A and B). We analyzed *Idh1^R132H^ Kat2a^Flox/WT^* (*Idh^mut^ Kat2a*HET) and *Idh1^R132H^ Kat2a^Flox/Flox^* (*Idh^mut^Kat2a*NULL) animals 4 and 20 weeks after Cre induction, to identify early and progressed *Idh1^R132H^* preleukemia states. Analysis of BM stem and progenitor composition revealed no differences between genotypes or time points (fig. S2, C to G). We did not observe differences in spleen or liver preleukemia burden (fig. S2, H and I). However, *Idh^mut^ Kat2a*NULL samples had a significant advantage in CFC replating in early preleukemia (4 weeks) (Fig. 1B), which was not sustained at the 20-week time point. This could be compatible with earlier selection of preleukemia cells upon *Kat2a* loss, which is achieved later in *Idh^mut^ Kat2a*HET animals as the *Idh1^mut^* phenotype progresses (Fig. 1C).

In an attempt to understand whether the early replating advantage in vitro could lead to accelerated leukemia development in vivo in the absence of other genetic events, we transplanted BM cells from *Idh^mut^ Kat2a*HET and *Idh^mut^ Kat2a*NULL mice, into irradiated CD45.1 recipients and followed them up for 1 year. Similar to single *Idh1^mut^* animals, we could not detect signs of leukemia development in transplanted mice (fig. S3A). Transplants showed accumulation of granulocyte-monocyte progenitor (GMP)–like (Lin-Kit^+^Sca1^−^FcγR^+^) donor cells, compatible with myeloproliferation (Fig. 1, D and E), which was identical between genotypes. Peripheral blood counts (fig. S3, B to D) and spleen and liver weights (fig. S3, E and F) were also similar. However, we observed the infiltration of the spleen and liver in one of three *Idh^mut^ Kat2a*NULL recipients, which was not present in *Idh^mut^ Kat2a*HET grafts (fig. S3G). Notably, *Idh^mut^ Kat2a*NULL cells showed enhanced colony-replating potential relative to *Idh^mut^ Kat2a*HET, which was comparable to that of BM from rare *Idh^mut^* leukemic animals (Fig. 1F). *Idh^mut^ Kat2aNULL* cells in CFC assays were enriched in c-Kit^+^Mac1^−^ cells (Fig. 1G) compatible with hindered differentiation and/or expansion of self-renewing cells. Overall, the results suggest that loss of *Kat2a* imparts leukemogenic properties to *Idh1^mut^* cells but is in itself not sufficient to drive leukemogenesis in the absence of additional cooperating genetic events.

### Loss of *Kat2a* accelerates *RUNX1-RUNX1T1* preleukemia–to-leukemia progression

**Fig. 2.**
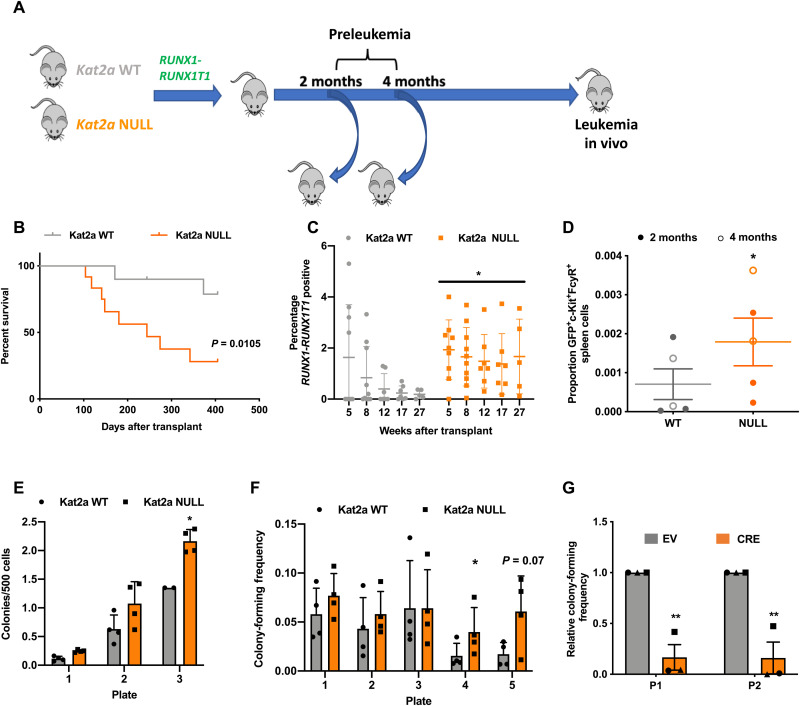
*Kat2a* loss accelerates *RT1*(*9a*) preleukemia to leukemia progression. (**A**) Experimental design. (**B**) Survival curve of *RT1*(*9a*) *Kat2a*WT and *Kat2a*NULL Kit^+^ BM recipients. *n* = 12 animals per genotype. *P < 0.05, log-rank test. (**C**) Quantification of peripheral blood green fluorescent protein (GFP) for animals in (A); GFP reports *RT1*(*9a*). Mean ± SD, *n* = 10 animals/genotype (8 weeks). *P < 0.05, two-way analysis of variance (ANOVA). (**D**) Flow cytometry analysis of *RT1*(*9a*) *Kat2a*WT and *Kat2a*NULL graft spleen cells 2 and 4 months after transplantation. Mean ± SD, *n* = 5. (**E**) CFC assay of *RT1*(*9a*) *Kat2a*WT and *Kat2a*NULL graft BM cells 4 months after transplantation. Mean ± SD, *n* = 4. (**F**) In vitro transformation of *Kat2a*WT and *Kat2a*NULL Lin^−^/Kit^+^ BM cells transduced with *RT1*(*9a*) retrovirus tested in CFC serial replating. Mean ± SD, *n* = 4. (**G**) CFC replating (plate = P1 and P2) analysis of *RT1*(*9a*) *Kat2a^Flox/Flox^* *Cre*^−/−^Kit^+^/Lin^−^ BM cells excised in vitro by lentiviral-delivered *Cre* recombinase [versus EV (empty vector)] after three rounds of colony replating. Mean ± SD, *n* = 3. All other analyses two-tailed *t* test, *P < 0.05 and **P < 0.01.

We next tested the impact of *Kat2a* loss on the preleukemia model driven by the exon 9a splicing variant of the *RUNX1-RUNX1T1*[*RT1*(*9a*)] fusion gene (*17*), which, when retrovirally delivered to adult BM cells, leads to long-latency, incomplete-penetrance leukemia in irradiated recipients (*19*–*20*). Using our previously described *Kat2a^Flox/Flox^ Mx1-Cre* mice (*12*), we isolated progenitor-enriched BM cells after *pIpC*-induced locus excision (fig. S4A) and delivered the *RT1(9a)* construct by retroviral transduction, as described (*20*). In all experiments, *Kat2a^Flox/Flox^Mx1-Cre^+/−^* (*Kat2a*NULL) cells were compared with *Kat2a^Flox/Flox^ Mx1-Cre^−/−^* (*Kat2a*WT) cells. We started by evaluating leukemia development after transplantation of RT1(9a) *Kat2a*NULL and *Kat2a*WT BM cells (Fig. 2A). Loss of *Kat2a* led to a marked decrease in survival of RT1(9a) recipient animals, compatible with accelerated leukemia progression (Fig. 2B). *Kat2a*NULL leukemias had a nonsignificant trend toward higher white blood cell counts (fig. S4, B to D) and spleen leukemia burden, with minimal infiltration of other organs (fig. S4, E to G). The surface phenotype of the leukemias was not different between genotypes, with the majority of Kit^+^ progenitor cells also Sca1^−/low^CD34^−^ (fig. S4H), as described (*17*). Analysis of early time points after transplantation showed that RT1(9a) engraftment became quickly fixed in the absence of *Kat2a* (Fig. 2C). *Kat2a*NULL/*RT1*(*9a*) cells obtained from healthy presymptomatic recipients were mildly enriched for Kit^+^FcgR^+^ cells (Fig. 2D) and displayed enhanced colony formation (Fig. 2E), compatible with accelerated preleukemia development. Similarly, *Kat2a*NULL cells directly tested in CFC assays upon retroviral transduction displayed enhanced replating potential. (Fig. 2F). In contrast, excision of *Kat2a* in *RT1(9a)* cells after in vitro transformation by three rounds of serial replating led to a reduction in colony formation (Fig. 2G), suggesting that *Kat2a* loss favors leukemia development only at a preleukemia stage. These latest observations mirror our previously identified role for *Kat2a* in maintenance of established leukemia stem-like cells and suggest that *Kat2a* plays stage-specific roles during leukemogenesis, which are preserved across leukemia models.

### Loss of *Kat2a* results in preleukemia cellular diversification

We had previously associated *Kat2a* function in LSC maintenance with stability of transcriptional programs (*12*). Using scRNA-seq, we showed that *Kat2a* loss resulted in diversification and branching of differentiation trajectories and associated with enhanced transcriptional noise, particularly in biosynthetic programs (e.g., ribosomal biogenesis and translation). We asked whether similar mechanisms were at play in preleukemia progression facilitated by *Kat2a* loss. We hypothesized that enhanced transcriptional variability leading to program diversification might increase the probability of accessing or seeding leukemia programs, resulting in the observed acceleration in leukemia progression.

We performed scRNA-seq analysis of preleukemia cells on the 10X platform, comparing transcriptional landscapes of *Kat2a*NULL and *Kat2a*WT *RT1(9a)* asymptomatic animals obtained 2 and 4 months after transplantation. We sequenced a total of 1767 cells sorted as *RT1(9a)*/GFP^+^Kit^+^ stem/progenitor and retrieved an average of 174,770 aligned reads per cell, corresponding to medians of 5939 unique molecular identifiers (UMIs) and 1575 genes per cell (Supplementary File 1). Less than 0.2% of reads aligned to mitochondrial DNA, denoting successful sequencing. Preprocessing steps are detailed in Materials and Methods.

**Fig. 3.**
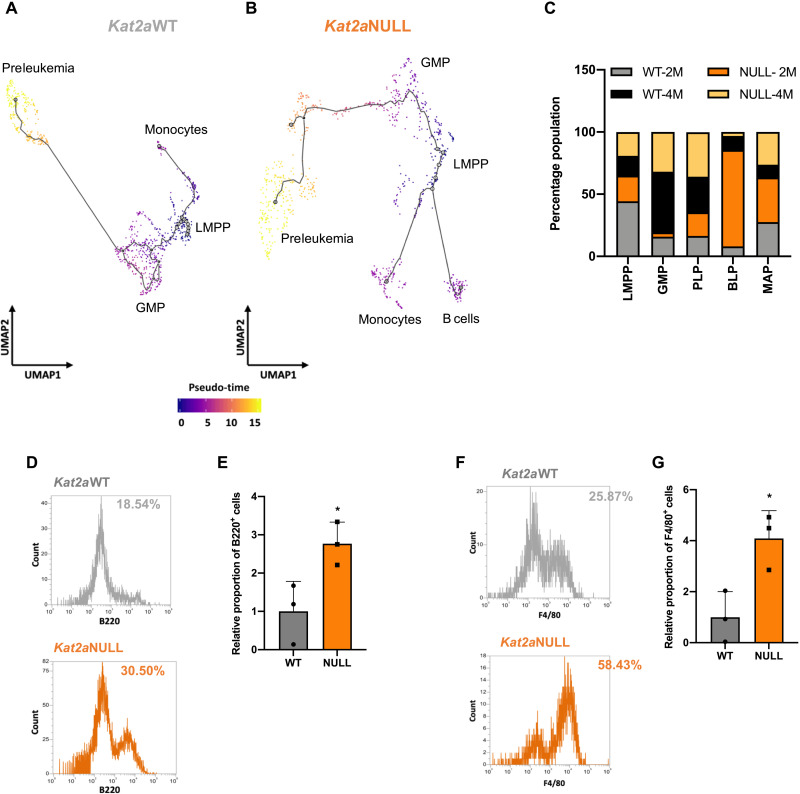
Loss of *Kat2a* diversifies cell fates and promotes RT1(9a) preleukemia progression. (**A** and **B**) Pseudo-time single-cell trajectory of (A) *Kat2a*WT cells (B) *Kat2a*NULL RT1(9a) cells 2 and 4 months after transplantation. Trajectories were inferred using Monocle3 (*21*); compartments were labeled as per hematopoietic markers in (fig. S5, C and D). (**C**) Proportion of candidate progenitor cell compartments in the RT1(9a) pseudo-time trajectory contributed by individual *Kat2a*WT or *Kat2a*NULL, 2-month or 4-month samples. (**D**) Representative flow cytometry histograms of B220 B cell marker detection in plate 2 CFC of *RT1(9a)*-transduced *Kat2a*WT and *Kat2a*NULL cells during in vitro transformation. (**E**) Aggregate results of B220 staining as in (D). Mean ± SD, *n* = 3. (**F**) Representative flow cytometry histograms of F4/80 monocyte marker detection in plate 2 CFC of *RT1(9a)*-transduced *Kat2a*WT and *Kat2a*NULL cells during in vitro transformation. (**G**) Aggregate results of F4/80 staining as in (F). Mean ± SD, *n* = 3.

We used the pseudo-time alignment algorithm Monocle (*21*) to understand the presence of different subpopulations of preleukemia cells and to infer relationships between those subpopulations along putative transformation trajectories, within and across early time points of transformation. Monocle uses a reverse graph embedding algorithm to align individual cells along a differentiation trajectory defined by progressive changes in their gene expression profile. Upon learning overall gene expression–based trajectory, each cell is sequentially positioned along the trajectory path to infer dynamic mechanisms of cell state transitions. We considered *Kat2a*WT and *Kat2a*NULL separately (Fig. 3, A and B, and fig. S5, A and B) to identify differences in transcriptional states that accompany loss of *Kat2a* and may help explain *Kat2a*NULL advantage in leukemia progression. We used transcripts of cell surface markers routinely used (*12*, *22*–*23*) for hematopoietic cell immunophenotyping to map the identity of cells along the pseudo-temporal trajectories (fig. S5, C and D). All cells were sorted as RT1(9a)^+^Kit^+^, thus capturing progenitor compartments. Cells at the origin of the trajectory expressed high *Ly6e* (*Sca1*), *Cd34*, and *Flt3*, compatible with lymphoid-myeloid primed progenitors (LMPPs) (*22*). LMPP-like cells were relatively enriched at 2 months after engraftment (Fig. 3E and fig. S5E), aligning temporal with pseudo-temporal trajectories. Accordingly, we did not find phenotypic LMPP-like cells in fully developed leukemias (fig. S6, A and B). LMPP-like cells were adjacent to an *Ly6e*^low^*Cd34*^+^*Fcgr3*^+^ state (fig. S5, C and D), compatible with GMPs (*23*). Unlike LMPP-like, GMP-like cells are more abundant at 4 months (Fig. 3C), suggesting cellular and temporal trajectory progression. Kit^+^Sca1^−/low^CD34^+^FcgR^+^ cells are variably represented in full-blown *RT1(9a)* leukemia (figs. S4H and S6, A and C). Additional states were represented in the trajectories. Two states, (1) and (2), diverged from LMPP-like cells in a distinct direction to GMP-like cells, with similar pseudo-times. The third state (3) denotes a later step in differentiation pseudo-time and follows the GMP-like path of the trajectory, with greater abundance of intermediate states upon *Kat2a* loss.

State (1) comprised *Ly6e*^+^*CD79a*^+^*Cd14*^−^ cells, which exhibit B lymphocyte–associated signatures (Supplementary File 3 and fig. S6D) and were designated B cell–affiliated progenitors (BAPs). These cells were more abundant in *Kat2a*NULL 2 months after transplantation but could also be observed at 4 months, in both genotypes (Fig. 3C and fig. S5E). Surface phenotyping of *Kat2a*NULL versus *Kat2a*WT cells undergoing in vitro transformation upon *RT1*(*9a*) transduction and serial replating independently confirmed enhanced specification of B220^+^ B lymphoid cells in *Kat2a*NULL samples (Fig. 3, D and E). Enhanced B lymphoid specification was transient, both transcriptionally (Fig. 3C) and cellularly (Fig. 3, D and E), and the phenotype also had reduced representation in fully developed leukemias (fig. S5E). State (2) comprised *Ly6e*^+^*Fcgr3*^+^*Cd14*^+^ cells, which have a monocytic/macrophage transcriptional affiliation (Supplementary File 4 and fig. S6E) and were dubbed monocyte-affiliated progenitors (MAPs). They could be observed in both genotypes, at both time points, with an enrichment in *Kat2a*-depleted samples (Fig. 3C and fig. S5E). Similar to B cell–affiliated cells, we captured more frequent emergence of F4/80^+^ monocyte-macrophages in *Kat2a*NULL samples undergoing in vitro transformation (Fig. 3, F and G), functionally confirming lineage diversification upon *Kat2a* knockout. MAP-like cells are minimally represented in fully developed *RT1(9a)* leukemia (fig. S6, A and B).

**Fig. 4.**
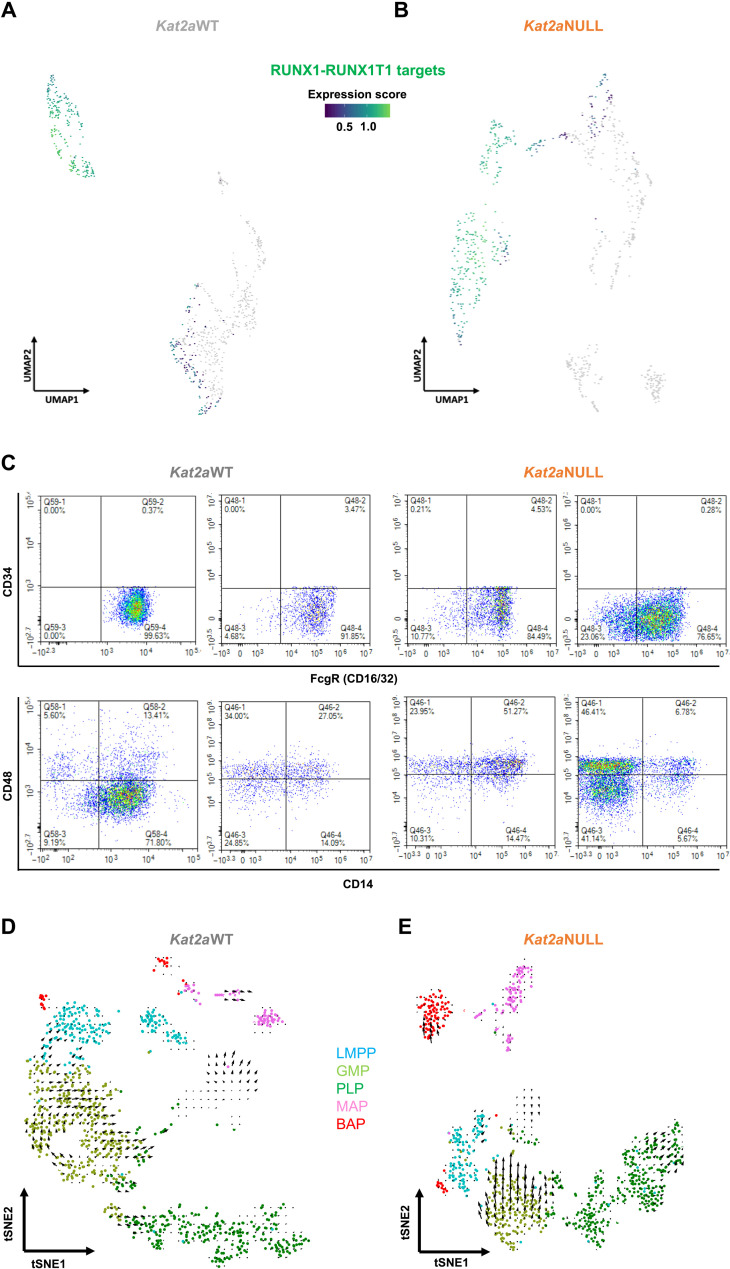
The PLP compartment captures RT1(9a) early transformed cells. (**A** and **B**) Expression of RUNX1-RUNX1T1 chromatin immunoprecipitation sequencing targets (*18*) in (A) *Kat2a*WT cells and (B) *Kat2a*NULL *RT1(9a)* single-cell trajectories. (**C**) Flow cytometry analysis of *RT1(9a)* pseudo-time–associated hematopoietic cell surface markers in representative Kat2a WT and Kat2a NULL AML. Plots are gated on RT1(9a)/GFP^+^Kit^+^Sca1^−^CD34^−^PLP-like cells (see fig. S4H). (**D** and **E**) RNA velocity (*25*) plots of RT1(9a) preleukemia in the presence (D) and in the absence (E) of *Kat2a*. Color coding reflects the progenitor compartments defined in Fig. 3. tSNE, *t*-distributed stochastic neighbor embedding.

The last cell state (3) was characterized as *Ly6e^low/−^Cd34^−^*, with variable levels of *Fcgr* and *Cd14* and rare detection of *Cd48* (Fig. 3, C and D). *RT1(9a)* leukemic progenitors were originally described as Kit^+^Sca1^−/low^CD34^−^FcgR^low^ (*17*), compatible with this last cell state. Accordingly, we detected expression of RT1 gene targets (*24*) specifically in this compartment (Fig. 4, A and B), suggesting development of a leukemogenic program. Inspection of the surface profile of *RT1(9a)* AML samples confirmed that most Kit^+^ cells were negative for Sca1 and CD34 (fig. S4H). *RT1(9a)* Kit^+^Sca1^−/low^CD34^−^ AML cells exhibited variable combinations of FcgR, CD48, and CD14 (Fig. 4C), suggesting that leukemias may have developed from different subclones within that compartment. We refer to this *Ly6e^low/−^Cd34^−^* state as preleukemia progenitors (PLPs). We sought to confirm the differentiation alignment of the different cell states using RNA velocity (Fig. 4, D and E) (*25*). The algorithm infers differentiation trajectories on the basis of relative representation of unspliced and spliced transcript variants, and the latter inferred as estimates of the future status of the cells over a relatively fast time frame. The greater distances observed in Monocle to BAP and MAP, and in opposite direction to PLP, are captured by scarcity of intermediate velocities that nevertheless recapitulate the Monocle directionality. Directionalities within the GMP-like and PLP states are better defined, particularly in *Kat2a*NULL cells. Significantly, *Kat2a*NULL cells (Fig. 4E) show increased velocity, which is unique within the PLP compartment, and may correspond to the more evenly populated trajectories within the *Kat2a*NULL Monocle trajectory (Fig. 3B).

### Loss of *Kat2a* increases transcriptional variability and destabilizes ribosomal biogenesis programs

**Fig. 5.**
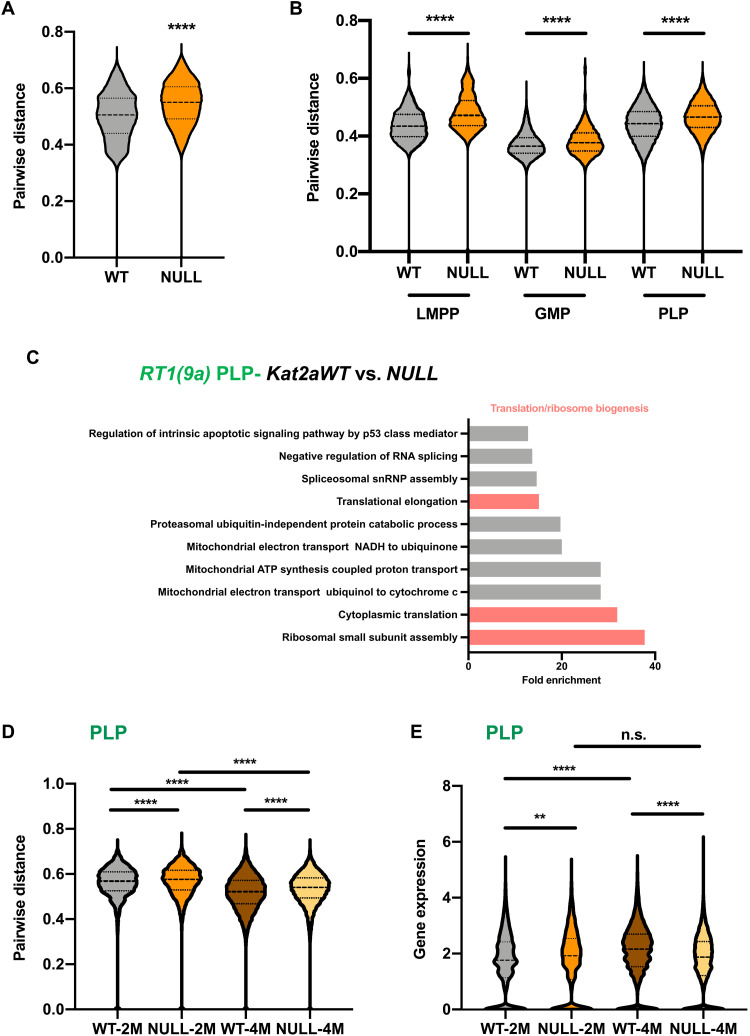
Loss of *Kat2a* destabilizes expression of ribosomal biogenesis and translation-associated genes. (**A**) Pairwise distance transcriptional variability measure (*27*) of *Kat2a*WT and *Kat2a*NULL *RT1(9a)* cells; top 500 most variable genes/genotype calculated by distance to the median CV (DM). ****P < 0.0001, nonparametric Kolmogorov-Smirnov (KS) test of cumulative distributions. (**B**) Comparison of *RT1(9a)* *Kat2a*WT and *Kat2a*NULL genotype-specific pairwise distances within individual LMPP, GMP, and PLP compartments. NULL up in all comparisons, with ****P < 0.0001, KS test. (**C**) Overrepresented gene ontology categories for genes down-regulated in RT1(9a) *Kat2a*NULL versus *Kat2a*WT PLP. *P-adj < 0.05. snRNP, small nuclear ribonucleoprotein; NADH, reduced form of nicotinamide adenine dinucleotide; ATP, adenosine 5′-triphosphate. (**D**) Pairwise distance of RT1(9a) PLPs. Comparisons consider correlations between ribosomal biogenesis genes, ****P < 0.0001, KS test. NULL up in all comparisons with WT; 2 months up in comparison with 4 months. (**E**) Distribution of expression levels for gene signatures in (C). **P-adj < 0.01, ****P-adj < 0.0001, two-tailed *t* test; n.s., not significant. NULL up at 2 months and down at 4 months in comparison with WT; WT up at 4 months relative to 2 months; comparison of NULL time points not statistically significant.

Given the previously established association between KAT2A and transcriptional noise regulation (*12*, *14*, *26*), we asked whether the cellular diversification and higher trajectory velocities observed upon *Kat2a*NULL loss were accompanied by, and putatively attributable to, enhanced variability in transcription. Pairwise distance (*27*) defines highly variable genes on the basis of a mean expression-corrected coefficient of variation or distance to the median (DM) (*28*) and inverts gene-to-gene correlations to estimate dispersion or distance in gene expression programs. Pairwise distance has been used as a measure of global transcriptional variability, or noise (*27*). Perhaps expectedly, given the differential diversity of cell types observed between *Kat2a*NULL and *Kat2a*WT *RT1(9a)* preleukemias, global pairwise distance was increased in *Kat2a*NULL cells (Fig. 5A), putatively capturing cellular heterogeneity. However, the same gain in pairwise distance was observed in the individual cell states (Fig. 5B), suggesting that loss of *Kat2a* may affect transcriptional variability. GMP-to-PLP transition is also accompanied by enhanced transcriptional variability (Fig. 5B), supporting the notion that *Kat2a* loss may facilitate preleukemia progression through enhanced transcriptional noise. To understand the nature of the transcriptional programs perturbed upon (i) *Kat2a* loss and (ii) preleukemia progression, we performed differential gene expression analysis of the scRNA-seq dataset. Comparison of *Kat2a*NULL to *Kat2a*WT cells revealed minimal changes in gene expression levels (fig. S7A), which were of down-regulation, as previously observed upon *Kat2a* loss (*12*). Consistent with our published data (*12*), differentially expressed genes between genotypes predominantly associated with ribosomal assembly and translation ontologies (fig. S7B and Supplementary File 5), a pattern particularly prominent within PLP (Fig. 5C and Supplementary File 6). The same ontologies were specifically down-regulated in *Kat2a*WT *RT1(9a)* PLPs compared to other cell states (fig. S7C and Supplementary File 7), capturing a reported decrease in protein synthesis in *RT1* leukemia (*29*). Ribosomal and translation ontologies (fig. S7, D and E, and Supplementary File 8) were also down-regulated in *Idh1^R132H^* mice. Together, our findings suggest a specific association of attenuated ribosomal programs with preleukemia progression, which may be further facilitated by *Kat2a* loss. *Kat2a* loss increases variability of ribosomal biogenesis programs in PLPs (Fig. 5D), which are themselves more variable than GMPs for the same programs (fig. S7F), suggesting enhanced noise at the transition (Supplementary File 9). The gene expression range in *Kat2a*NULL PLPs favors lower mean values (Fig. 5E), specifically at 4 months. In support of the functional impact of the transcriptional perturbation, *Kat2a* loss results in decreased protein synthesis (fig. S7, G and H).

### Reduced protein synthesis activity transiently facilitates preleukemia progression

**Fig. 6.**
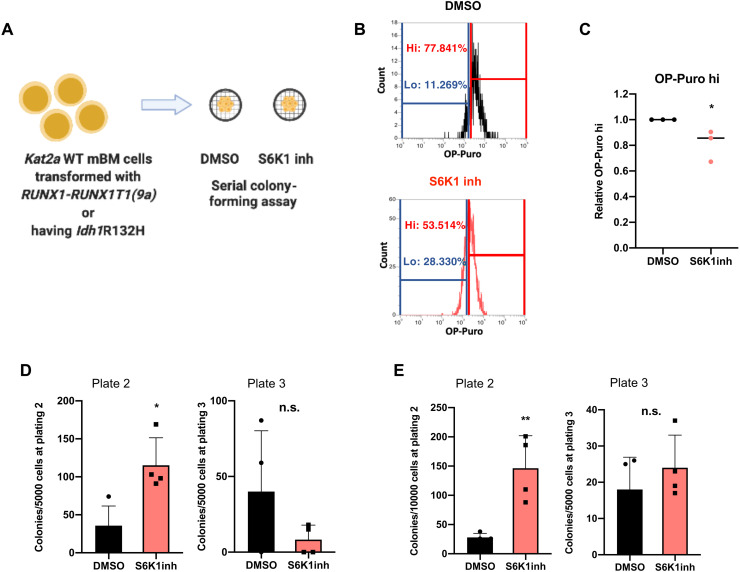
Inhibition of protein synthesis phenocopies effects of *Kat2a* loss facilitating preleukemia transformation. (**A**) Schematic of S6K1 inhibition assays. mBM, mouse BM. (**B**) Representative OP-Puro incorporation flow cytometry of S6K1inh-treated *RT1(9a)* *Kat2a*WT cells. OP-Puro high cells, hi; OP-puro; low cells, lo. (**C**) Quantification of OP-Puro high (hi) cells in (B), relative to dimethyl sulfoxide (DMSO). Mean ± SD, *n* = 3. *P < 0.05, two-tailed *t* test. (**D**) CFC replating of *Kat2a*WT *RT1(9a)* in vitro transformation in the presence of S6K1inh (control, DMSO). Plate 2 (left): means ± SD, *n* = 4, *P < 0.05. Plate 3 (right): mean ± SD, *n* = 4, n.s., two-tailed *t* test. (**E**) (D) CFC replating of *Idh1^R132H^* *Kat2a*WT cells in the presence of S6K1inh (or DMSO), 4 weeks after locus activation. Plate 2 (left): mean ± SD, *n* = 4, **P < 0.01. Plate 3 (right): mean ± SD, *n* = 4, n.s., two-tailed *t* test.

We tested the contribution of reduced protein synthesis activity to preleukemia progression by treatment with the S6K1 inhibitor (S6K1inh) PF4708671 (Fig. 6A), which impairs protein synthesis activity confirmed by reduced *O*-propargyl-puromycin (OP-Puro) incorporation in nascent peptide chains (Fig. 6, B and C). We treated *Kat2a*WT *RT1(9a)* cells with S6K1inh and tested their leukemia transformation potential in vitro through CFC assay replating. S6K1-inhibited cells displayed enhanced colony formation upon replating (Fig. 6D), suggesting a contribution to leukemia transformation. However, the increase in colony formation was transient and eventually lost upon subsequent replating (Fig. 6D). This suggests that the effects of reduced protein synthesis on leukemia cells may vary with progression of transformation, reconciling our data with prior analysis of established *MLL-AF9* cells, in which reduced OP-Puro incorporation associated with *Kat2a*NULL-mediated extinction of LSCs (*12*). We observed a similar pattern of transient increase in colony formation of *Idh1^R132H^* preleukemia cells treated with S6K1inh (Fig. 6E). Together, the data suggest that reduced ribosomal assembly and protein synthesis facilitate preleukemia progression. Exploration of lower levels of expression of translation-associated genes as a consequence of enhanced transcriptional variability may be instrumental in the acceleration of preleukemia to AML transition upon *Kat2a* loss. As leukemia progresses, variability in ribosomal biosynthesis programs may become attenuated with deviation from an optimal level no longer favorable to transformation.

## DISCUSSION

**Fig. 7.**
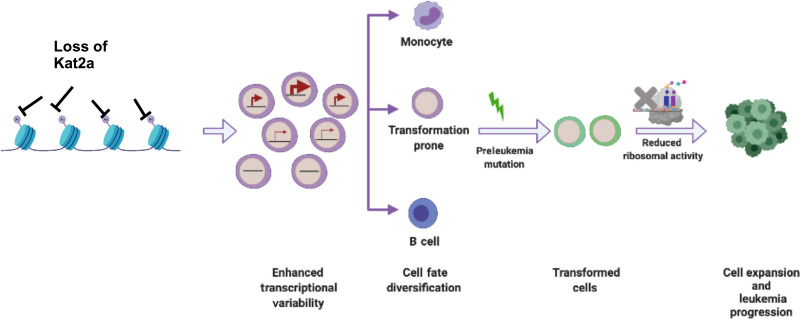
Working model of the role of Kat2a loss in preleukemia progression. Kat2a loss facilitates generation or selection of cell types susceptible to transformation. Preleukemia progression is further aided by variability in ribosomal protein gene transcription and reduced protein synthesis.

In this study, we have shown that *Kat2a* loss facilitates preleukemia progression in *Idh1^R132H^* and *RUNX1-RUNX1T1*(*9a*) mouse models of human disease, with acceleration of frank leukemia onset in the case of *RT1(9a)*. Loss of *Kat2a* resulted in enhanced variability of transcription, leading to diversification of cell fates, including accumulation of PLP cells. In the context of an early genetic event such as *RT1*(*9a*) or *Idh1^R132H^*, which do not allow for full leukemia transformation, the cellular heterogeneity that ensues creates the opportunity for specification and expansion of transformation-prone cells, on which additional molecular events may act to progress the leukemic process (Fig. 7).

*RT1* progenitor cells have been variably characterized as Kit^+^Sca1^+^ cells (*17*) and Kit^+^Sca1^−^FcgR^+^ GMP-like cells (*19*), with the (9a) variant denoting a Kit^+^Sca1^−^CD34^−/low^FcgR^−/low^ phenotype (*17*). The variability in cellular composition is notable between individual animals (fig. S5) and likely denotes the contribution of additional mutations to the establishment of full-blown leukemia (*30*), which may lose dependence on the presence of *RUNX1-RUNX1T1* (*29*) and become sensitive to its level of expression (*31*, *32*). Cellular variability may emerge as a downstream consequence of different additional genetic events or reflect differential upstream vulnerability to specific mutations. Loss of *Kat2a* may facilitate the latter process. By destabilizing transcription and diversifying cellular output as one consequence of moving stem and progenitor cells out of their status quo, *Kat2a*NULL animals may generate additional types of *RT1(9a)* translocation-carrying cells able to respond to downstream mutations and/or be transformed by them. It is unlikely that *Kat2a* loss itself contributes to the genetic load. *Kat2a*NULL animals are not at a risk of myeloproliferation (*12*), and no recurrent *KAT2A* mutations have been described to date in association with hematological or solid cancers. In contrast, *Kat2a* ablation consistently changes cellular composition (*12*–*14*, *33*), making it a more likely facilitator event to generate “second hit”–responsive preleukemia cells. Future studies combining genetic barcoding and phenotyping on a time course of transformation should provide definitive evidence of such an effect.

Further to or concomitantly with cellular diversification and putative differential susceptibility to secondary genetic hits, the molecular programs affected by *Kat2a* loss can also contribute to the leukemogenic process. Ribosome biosynthetic and translation factor genes are pervasive targets of KAT2A (*12*, *33*), and our data suggest that destabilization of translation acts to facilitate transformation at least transiently and down-regulation of translation-associated genes may accompany preleukemia to leukemia progression. Enhanced transformation may be achieved by surveying and selection of biosynthetically quiescent cell states, which evade further diversification and respond to additional mutations with disease propagation and progression. Inspection of noise-responsive programs in chronic lymphocytic leukemia has captured ribosome biogenesis and translation as a significant prognostic module (*34*), and loss of ribosome biosynthetic activities plays a role in T acute lymphoblastic leukemia progression (*35*). The latter study also implicated decreased mitochondrial metabolic activity, which we have shown to be responsive to *Kat2a* loss, in leukemia development.

However, established leukemia cells can be dependent on active translation for their maintenance (*36*), and AML subtypes, namely, those with *RUNX1* mutations (*37*), are therapeutically sensitive to inhibition of protein synthesis. Despite the contribution of reduced or perturbed translational activity to transformation, our results suggest that the effect is transient, and we had previously observed that the inhibition of protein synthesis reduced colony formation in established *MLL-AF9* leukemia cells (*12*). Accordingly, *MLL-AF9* leukemia knockout for *Kat2a* displayed enhanced noise specifically in translation-associated genes, which was accompanied by reduced protein synthesis and associated with depletion of leukemia stem-like cells (*12*). It is possible that fully transformed, well-adapted leukemia cells buffer transcriptional variability to maintain stable self-renewal signatures and optimal biosynthetic, translation rates. In this context, instability of transcriptional programs may shift biosynthetic homeostasis and perturb cellular identity and mal-adapt leukemia stem-like cells, with antileukemia effects. Thus, stage-specific tuning and untuning of transcription and translation may be used to modulate cancer progression, a principle that can be extended to other cancer state transitions such as metastasis or drug resistance with prognostic and therapeutic potential.

## MATERIALS AND METHODS

### Preleukemia mouse models

Mice were kept in a specific pathogen–free animal facility, and all experimental work was carried out under U.K. Home Office regulations. Animal research was regulated under the Animals (Scientific Procedures) Act 1986 Amendment Regulations 2012 following ethical review by the University of Cambridge Animal Welfare and Ethical Review Body. Peripheral blood was collected by saphenous vein, and differential blood cells counts were determined using a Vet abc automated counter (Scil Animal Care, Viernheim, Germany).

### Generation of an *Idh1^R132H^* mouse model

Targeting vector was generated as follows using methods described previously (*38*). The WT *Idh1* locus (endogenous *Idh1* sequence, including arms of homology, was captured by gap repair as previously described). The following primer pairs were used to amplify the “U” cassette containing attR1 and attR2 gateway cloning sites containing a *Zeo*/*PheoR* selection cassette with the appropriate overhangs to allow insertion of this cassette between exons 2 and 3 of the *Idh1* locus by recombineering (Table 1).

**Table 1.** Primers for genotyping.

**Target**	**Forward primer (5′-3′)**	**Reverse primer 1 (5′-3′)**	**Reverse primer 2 (5′-3′)**
*Mx1-Cre*	CGTACTGACGGTGGGA GAAT	TGCATGATCTCCGGT ATTGA	–
*Kat2a*	CACAGAGCTTCTTGGA GACC	GGCTTGATTCCTGTA CCTCC	–
*Idh1*	GTTGGTGGATTCCATTGCTT	TGTTAGTCCCAACCC CTTCC	GACAAACTGACAGGCTG CAA
Amplification of U cassette	AAGTCCAACCTTATTGTCCCATCATAAGTTTTATA CTCTGTAAGTAATGACCGCCTACTGCGACTATAGA	AGGTTCACCCTATGACTAACTGGCTCTAACAAAAG AGTTCTCAGCTCTTTAAGGCGCATAACGATACCAC	–
Amplification of G cassette	GCAATAGGAACCCTTTGCCATACTTAATTTTACTTCC ATAAATCTCAAGTTCCTGTGTGAAATTGTTATCCGC	ACAAACTAGCTAACCTGATGGATGCAGTAATGAGT AACACAGGAGATCCTCCACTGGCCGTCGTTTTACA	–
WT control assay	GGGCTAGGGGAAGCGCCATC	TGCGCAGGCCAAAAGCCCAT	–
5′ integration	TGGCTGGAAAACAAAAAGATCGG	CGTTATGCGCCTTAAAGAGCTGA	–
3′ integration	TGGATCCGGGAAGTTCCTATTCC	TGGCACAGGCACAGAGGGA	–
3′ internal probe	GGAGTGTTGTATCGCAGCAA	GCGCTAGGATTAAAGGCACA	–
5′ internal probe	TCAGCATTCCCTAGGCACAA	TCTCTTGAGTGTGAGGCCAG	–

The following primers were used to amplify the “G” cassette that contains attR3 and attR4 gateway sites flanking an *AmpR* cassette. Appropriate overhangs were incorporated into these primers to allow “gap repair” subcloning and retrieval of arms of homology 5′ and 3′ to exons 2 and 4 (5.9 and 3.6 kb, respectively) from the *Idh1* containing bacterial artificial chromosome (Table 1).

A custom gene block (GeneArt, Thermo Fisher Scientific) containing sequence encoding the mutant R132H substitution in exon 3 was cloned into the subsequent U/G-captured intermediate—replacing the WT exon3 by standard restriction enzyme cloning using Sna BI and Csp CI to generate an R132H mutant U/G vector. A custom cDNA flanked by Afl II and Asc I sites, containing AttL1-loxP-En2SA-Idh1 cDNA exons3 to 9–SV40 pA-loxP-FRT was synthesized (GeneArt, Thermo Fisher Scientific) and cloned into the PL1PL2 containing an FRT-flanked NeoR cassette. This generated the “SA-Idh1 exon 3-9 cDNA” cassette.

These vectors and the PL3L4 vector were combined in the downstream L/R clonase reaction to successfully generate the Idh1R132H-*NeoR*TV vector. All intermediate and final vectors were sequence-verified. The *Idh1^R132H-NeoRTV^* targeting vector was electroporated into mouse ES cells, and genotyping was performed for on target integration at the endogenous *Idh1* locus using a series of long-range polymerase chain reaction (PCR) reactions (Table 1):

Genomic DNA (gDNA) extracted from heterozygous-targeted single-cell mouse ES cell clones were subjected to Southern blot to confirm the structural integrity and confirmation of site-directed recombination of FRT and LoxP recombination sites, before microinjection. FRT recombination and removal of the *NeoR* cassette were mediated by expression of flippase via transient transfection of the pCAG-FlpO plasmid (Addgene, #89574). LoxP recombination and deletion of the “SA-Idh1 exons 3 to 9 cDNA” cassette was mediated by expression of Cre via transient transfection of the pCAG-Cre plasmid (Addgene, #13775). gDNA extracted from single-cell clones were subjected to Southern blot to confirm the successful removal of the *NeoR* resistance cassette and LoxP recombination to generate the *Idh1^R132H-TV^* and *Idh1^R132H-KI^* alleles, respectively (fig. S1A). The primers that were used to generate Southern blot hybridization probes for the respective 3′ internal (FLP assay) and 5′ internal (Cre assay) are mentioned in Table 1.

Positively targeted heterozygous clones were selected for microinjection. Chimeric offspring were then selected for downstream breeding and germ line transmission of the *Idh1^R132H-NeoRTV^-*targeted allele.

The FRT-flanked neomycin-resistant cassette, used for positive enrichment of targeted mouse ES cells, was removed by breeding to FLPe mice [as previously described (*39*)]. F1 mice were backcrossed to WT C57Bl6 mice, and mice negative for the presence of the RosaFLPe transgene and positive for the inducible *Idh1Knock-In^R132H^* allele were selected for downstream cohort generation by subsequent crosses with the inducible Mx1-Cre transgenic mouse model (fig. S1C). Standard PCR genotyping for the WT and mutant alleles was performed (using the primers detailed in Table 1). Subsequent crosses to homozygous *Nras^G12D/G12D^* and *Npm1^cA/cA^* cohorts were used to generate experimental model cohorts, as described previously (*40*).

To generate a Mx1-Cre-inducible mouse with *Idh1^mut/WT^*- and *Kat2a*-floxed alleles, *Idh1^mut/WT^Kat2a^WT/WT^Mx1-Cre^+/−^* males were crossed with *Idh1^WT/WT^Kat2a ^fl/fl^Cre^−/−^* females. The first generation carried a *Kat2a ^fl/WT^* genotype (referred as *Kat2a* HET) with either *Idh1^WT/WT^* or *Idh1^mut/WT^* and *Mx1-Cre^+/−^*. *Idh1^WT/WT^Kat2a ^fl/WT^* and *Idh1^mut/WT^Kat2a ^fl/fl^* offsprings were crossed to obtain experimental genotypes referred to as *Kat2a* HET (*Idh1^mut/WT^Kat2a ^fl/WT^*) and *Kat2a*NULL (*Idh1^mut/WT^Kat2a ^fl/fl^*) maintaining an heterozygous *Mx1-Cre* allele.

### *Kat2a* conditional knockout model

*Kat2a^fl/fl^* conditional knockout mice have been previously described (*12*).

### Genotyping

Ear notch biopsies were digested overnight in lysis buffer [50 mM sodium chloride, 50 mM tris(hydroxymethyl)aminomethane hydrochloride, 5 mM ethylenediaminetetraacetic acid, 20% SDS, and proteinase K (0.5 mg/ml)] at 55°C and 750 rpm using a Thermo-Shaker (BioSan). DNA extraction used isopropanol-based precipitation. Mice were genotyped using the primers in Table 1, following the PCR protocol: 95°C, 3 min, 40× [94°C, 30 s; 60°C, 30 s (57°C, 30 s for *Idh1*); 72°C, 90 s (30 s for *Idh1*)]; 72°C, 10 min. DNA products were run on a 1% agarose gel in TAE (Tris-acetate-EDTA) (1×), at 100 V and visualized using an AlphaImager ultraviolet transilluminator (Protein Simple). Cre-mediated recombination was induced in 8-week-old mice by administration of five–alternate day intraperitoneal injections of polyinosinic:polycytidylic acid (pIpC), 300 μg per dose. *Idh1* recombination was confirmed by PCR (Table 1, reverse primer 2) following the same PCR protocol.

### Preleukemia cell engraftment

BM cells were isolated from long bones of *Kat2a*WT and *Kat2a*NULL animals as described (*12*). Briefly, following red blood cell lysis, BM-nucleated cells were depleted of differentiated cells using a cocktail of biotinylated lineage (Lin) antibodies and streptavidin-labeled magnetic nanobeads (BioLegend), according to the manufacturer’s instructions. Lin-depleted cells pooled from four *Kat2a*WT or *Kat2a*NULL animals were cultured separately overnight at 37°C and 5% CO_2_ in RPMI supplemented with 20% heat-inactivated fetal bovine serum (FBS) (R20), l-glutamine (2 mg/ml), 1% prostate-specific antigen, murine interleukin-3 (mIL-3; 10 ng/ml), mIL-6 (10 ng/ml), and murine stem cell factor (mSCF; 20 ng/ml) (cytokines from PeproTech) (supplemented R20), followed by retroviral transduction.

Retroviral construct *MSCV-AML1/ETO-IRES-GFP* was previously described (*20*). Viral particle production and transduction of the genotype-specific pools were done as described previously (*12*). Green fluorescent protein (GFP) levels were assessed by flow cytometry. One million BM cells obtained after transduction of *Kat2a*WT and *Kat2a*NULL pools were injected per animal into >8-week-old, C57/BL6 mice, which were lethally irradiated [2 × 5.5 gray (Gy)]. Seventeen mice per group were injected for preleukemia and leukemia studies. Leukemia survival studies were performed as two independent experiments. Leukemic mice were collected on the basis of symptoms of hunched posture, inappetence, and lethargy.

BM cells obtained from *Idh1^R132H^*-transformed *Kat2a*HET and *Kat2a*NULL animals 20 weeks after pIpC were injected into CD45.1, C57/BL6 mice (*n* = 8 per group), which were sublethally irradiated (1 × 8 Gy). Bones and spleens were collected after 1 year of transplantation and analyzed by flow cytometry.

### CFC assays

For analysis of preleukemia samples from *RT1*(*9a*) and *Idh1^R132H^*, 50,000 BM cells were plated in MethoCult M3434 (STEMCELL Technologies), following the manufacturer’s protocols. Colonies were scored 7 to 10 days after plating. Cells were collected from plates, washed, dispersed to a single-cell suspension, and serially replated for transformation analysis.

In S6K1 inhibition studies, 10,000 *Kat2a*WT BM cells transduced with *RT1*(*9a*) or carrying the recombined *Idh1^R132H^* allele, were plated in MethoCult M3434 containing freshly added dimethyl sulfoxide (DMSO) (vehicle) or 10 μM PF4708671 (Tocris) with a final concentration of 0.1% DMSO. Colonies were scored as above.

### Leukemia maintenance in vitro

Pooled BM cells collected from two to three 12-week-old *Kat2a* floxed *Mx1-Cre*^−/−^ animals without pIpC treatment were retrovirally transduced with *RT1*(*9a*) and serially replated in MethoCult M3434 for a total of three platings (4000 cells per plating). At plate 3, cells were collected, transduced with a MIGR-Cre-OP-Puro (Cre^+^) or a MIGR-OP-Puro (empty) retrovirus, and cultured for 48 hours under puromycin selection, as described (*20*). Transduced and antibiotic-selected cells were assessed for colony formation over two rounds of plating in MethoCult M3434 (4000 cells per plating per condition) in the presence of puromycin. Colonies were scored 7 to 10 days after plating.

### Flow cytometry analysis

Cell surface analysis of mouse BM and spleen was performed as described (*12*), using the antibodies in Table 2. Where indicated in the text, the following gating strategies were used for quantification of hematopoietic stem and progenitor cell compartments: hematopoietic stem cell (HSC), Lin^−^cKit^+^Sca1^+^CD34^−^Flt3^−^; multipotent progenitor (MPP), Lin^−^cKit^+^Sca1^+^CD34^+^Flt3^−^; lympho-myeloid primed progenitor (LMPP), Lin^−^cKit^+^Sca1^+^CD34^+^Flt3^+^; common myeloid progenitor (CMP), Lin^−^cKit^+^Sca1^−^CD34^+/low^CD16/32^low^; granulocyte-monocyte progenitor (GMP), Lin^−^cKit^+^Sca1^+^CD34^+^CD16/32^high^; megakaryocyte–erythroid progenitor (MEP), Lin^−^cKit^+^Sca1^+^CD34^−^CD16/32^−^; Lin^−^, CD3e^−^B220^−^Gr1^−^CD11b^−^Ter119^−^.

**Table 2.** Antibodies used in flow cytometry analysis, cell sorting and lineage selection.

**Antibody**	**Fluorochrome**	**Catalog ID**	**Clone**	**Dilution**	**Supplier**
CD45R/ B220	APC-Cy7	103223	RA3-6B2	1:50	BioLegend
CD45R/ B220	PerCP-Cy5.5	103235	RA3-6B2	1:100	BioLegend
CD117/c-Kit	APC-Cy7	105826	2B8	1:50	BioLegend
CD117/c-Kit	BV785	105841	2B8	1:100	BioLegend
CD11b/Mac1	AF700	101222	M1/70	1:200	BioLegend
CD16/32/FcγR	PE	101308	93	1:100	BioLegend
CD16/32/FcγR	BV421	101331	93	1:200	BioLegend
CD34	APC	128612	HM34	1:100	BioLegend
F4/80	PE	123109	BM8	1:100	BioLegend
Gr1	PB	108430	RB6-8C5	1:100	BioLegend
CD14	PE-Cy7	123315	Sa14–2	1:200	BioLegend
CD48	BV510	103443	HM48–1	1:200	BioLegend
Sca1	PE-Cy7	108114	D7	1:100	BioLegend
Sca1	PerCP	108121	D7	1:100	BioLegend
CD45R/B220 (Lin)	Biotin	103204	RA3-6B2	1:300	BioLegend
Ter119 (Lin)	Biotin	116204	Ter119	1:300	BioLegend
Gr1 (Lin)	Biotin	108404	RB6-8C5	1:300	BioLegend
CD3e (Lin)	Biotin	100304	145-2 C11	1:300	BioLegend
CD11b (Lin)	Biotin	101204	M1/70	1:300	BioLegend
CD48	Biotin	103409	HM48-1	1:100	BioLegend
Streptavidin	BV421	405226	–	1:200	BioLegend
Streptavidin	BV605	405229	–	1:200	BioLegend
Streptavidin	APC-Cy7	405208	–	1:200	BioLegend
Nanobeads	Streptavidin	76447	–	1:10	BioLegend
Hoechst 33258	–	H3569	–	1:10,000	Invitrogen
Click-iT Cell Reaction Buffer Kit	AF647 azide	A10277	–	1:500	Invitrogen

### scRNA-seq preparation and analysis

Preleukemia BM samples were collected from individual animals engrafted with *RT1*(*9a*)-transduced *Kat2a*WT or *Kat2a*NULL pooled cells, 2 and 4 months after transplantation, and stored at −150°C. Cells were thawed, recovered in R20 medium, and sorted on an Influx sorter (BD) as Hoechst 33258-negative (live), GFP^+^ [*RT1*(*9a*) reporter], and cKit^+^ (early progenitors) singlets. Sorted cells were immediately used for library preparation with the Chromium Next GEM Single-Cell 3′ GEM, Library, and Gel Bead Kit v2 (10X Genomics). Libraries were quality-controlled and underwent paired-end sequencing on an Illumina NextSeq 500 Sequencer. Library preparation and sequencing were performed at Cancer Research UK (CRUK) Cambridge Research Institute. Raw single-cell RNA-seq fastq reads were analyzed using Cell Ranger software (v2.2) to obtain the cell-gene count matrix (Table 3). Preprocessing analysis yielded a gene-count matrix with 1675 cells in total with a median UMI count of 5939 and 1575 median genes per cell. The count-matrix data were preprocessed with Seurat v2.4 (*41*) as described (*12*). Each cell that expressed less than 500 genes was considered to be of poor quality and was filtered out. Differential gene expression was obtained with DESeq2 (*42*), as per the implementation in Seurat v2.4, for pairwise comparisons between genotypes, globally or at individual time points, between two individual cell compartments, or between one individual compartment and the remaining cells, e.g., for BAP- and MAP-associated signatures. For genes with adjusted *P* < 0.05, differential expression calculated using log_2_ fold change (FC) was deemed as significant where |log_2_ FC| > 0.26 (20% fold difference in averages). Gene ontology analysis was performed using Panther 14.0 (*43*), selecting Fisher’s exact test with Bonferroni correction. Transcriptional variability analysis used pairwise distance between gene correlations as a measure of cellular heterogeneity, by identifying the top 500 highly variable genes based on distance-to-median (DM) and calculating Spearman correlation coefficients between all gene pairs (*27*). Pseudo-time analysis was performed using Monocle v3.0 (*21*) separately for *Kat2a*WT and *Kat2a*NULL cells. Cell identities were attributed by inspection of the presence and level of transcripts of lineage-associated markers and transcription factors commonly used in classification of hematopoietic progenitors (see fig. S5, C and D) (*22*, *23*) and manual annotation of contiguous or discrete regions on the basis of dominant combinatorial marker expression. LMPP-like space, Ly6e^+^CD34^+^Flt3^+^CD79a^−^CD14^−^ (also Gata2^+^Myb^+^), was observed at the origin of the trajectories. Ly6e^+^CD34^+^Flt3^−^CD79a^−^CD14^−^FcgR^+^ (also Cebpa^+^), Ly6e^+^CD34^−^Flt3^−^CD79a^+^CD14^−^FcgR^−^ (also CD19^+^Il7r^+^Ebf^+^), and Ly6e^+^CD34^−^Flt3^−^CD79a^−^CD14^+^FcgR^+^ (also Mafb^+^) were GMP-like, B cell–affiliated, and monocyte/macrophage-affiliated, respectively. Uniform manifold approximation and projection (UMAP) plot regions were consistent between genotype-specific and global pseudo-time trajectories. RNA velocity analysis was performed using velocyto.py analysis pipeline (*25*). Raw fastq files were used to generate .loom files that were used for velocity calculation for individual genotypes and individual time points.

**Table 3.** Cell-gene count matrix specification.

**Total number of cells sequenced**	**1767**
Median number of genes per cell	1575
*Kat2aWT* 2 months	379
*Kat2aNULL* 2 months	369
*Kat2aWT* 4 months	518
*Kat2aNULL* 4 months	501

### Bulk RNA-seq analysis

Total RNA was extracted from mouse BM aspirates, following enucleated cell lysis. Paired-end RNA-seq reads were mapped to the mouse genome (UCSC mm10) using STAR with default parameters (*44*). The total number of reads aligning to the exons of each gene [as per GENCODE vM4 (*45*)] were counted using HTSeq count (*46*). Read counts were used for FPKM (Fragments Per Kilobase of transcript per Million mapped reads) computation and differential expression analyses using DESeq2 between sex-matched preleukemia and AML samples (*42*). Preleukemia samples analyzed were: *Idh1^R132H^* (*n* = 2), *Idh1^R132H^ Npm1c* (*n* = 2), *Npm1c N-Ras^G12D^ Idh1^R132H^* (*n* = 2), and *Idh1^WT^* (*n* = 6 female, 4 male); AML samples analyzed were: *N-Ras^G12D^ Idh1^R132H^* (*n* = 3) and *Npm1c N-Ras^G12D^ Idh1^R132H^* (*n* = 3).

### Measurement of protein synthesis

Protein synthesis rates were estimated using OP-Puro (Thermo Fisher Scientific) incorporation, as described (*12*). In detail, 1 million *RT1*(*9a*) *Kat2a*WT or *Idh1^R132H^ Kat2a*HET cells treated with 10 μM S6K1inh PF4708671 versus DMSO (vehicle) were collected from successive replating of colony-forming assays and cultured for 2 hours in the presence of cytokines, mSCF (20 ng/ml), mIL-3 (10 ng/ml), and mIL-6 (10 ng/ml) in R20 medium. In other assays, *Idh1^R132H^ Kat2a*HET versus *Kat2a*NULL BM cells collected 20 weeks after pIpC treatment for locus activation/excision were thawed and cultured overnight in the same conditions. A final concentration of 12.5 μM OP-Puro was added directly to 80% of each culture for the last hour of the culture period; the remainder 20% cells were treated with phosphate-buffered saline (PBS) and processed in parallel as control. After incubation, cells were washed with ice-cold PBS without Ca^2+^ or Mg^2+^ (Sigma-Aldrich) and resuspended in PBS/10% FBS for cell surface staining with c-Kit–APCC7, CD11b-biotin, and Gr1-biotin (all from BioLegend; see Table 2), followed by Streptavidin Brilliant Violet 605 (also from BioLegend), both staining steps for 30 min on ice. After washing, cells were fixed in 1% paraformaldehyde in PBS for 15 min on ice protected from light, washed, and permeabilized in PBS/3% FBS/0.1% saponin (permeabilization buffer) at room temperature, in the dark, for 5 min. Cells were washed and used immediately in the azide-alkyne cyclo-addition reaction with the Click-iT Cell Reaction Buffer Kit (Thermo Fisher Scientific, C10269) and Alexa Fluor 647–Azide (Thermo Fisher Scientific, A10277) with a master reaction solution freshly prepared for immediate use, as per the manufacturer’s instructions. Alexa Fluor 647–Azide was used at a final concentration of 5 μM. The reaction proceeded in the dark at room temperature for 30 min; cells were washed twice in permeabilization buffer and resuspended in PBS, 5 min before flow cytometry analysis.

### Statistical analysis

Experiments were performed in triplicate, with any exceptions specifically indicated in the text or figure legends. Data are plotted as mean ± SD with statistical tests described in the respective figure legends. Statistical analysis was performed using GraphPad Prism 8.0 software. R language was used for analysis of single-cell RNA-seq data.

## Supplementary Materials

### This PDF file includes:

Figs. S1 to S7 References

## Notes

View/request a protocol for this paper from *Bio-protocol*.
