# Supplementary material for: Transcriptional variability accelerates preleukemia by cell diversification and perturbation of protein synthesis
Source: Sci Adv. 2022 Aug 3;8(31):eabn4886. doi: 10.1126/sciadv.abn4886 (PMC9348803; doi:10.1126/sciadv.abn4886)
Supplement: Supplementary file 1 — Figs. S1 to S7 References [file sciadv.abn4886_sm.pdf]

Science Advances
AAAS

Supplementary Materials for

Transcriptional variability accelerates preleukemia by cell diversification and perturbation of protein synthesis

Shikha Gupta *et al.*

Corresponding author: Cristina Pina, cristina.pina@brunel.ac.uk

Sci. Adv. **8**, eabn4886 (2022)
DOI: 10.1126/sciadv.abn4886

The PDF file includes:

Figs. S1 to S7
Legends for supplementary files S1 to S9
References

Other Supplementary Material for this manuscript includes the following:

Supplementary Files S1 to S9

Fig S1

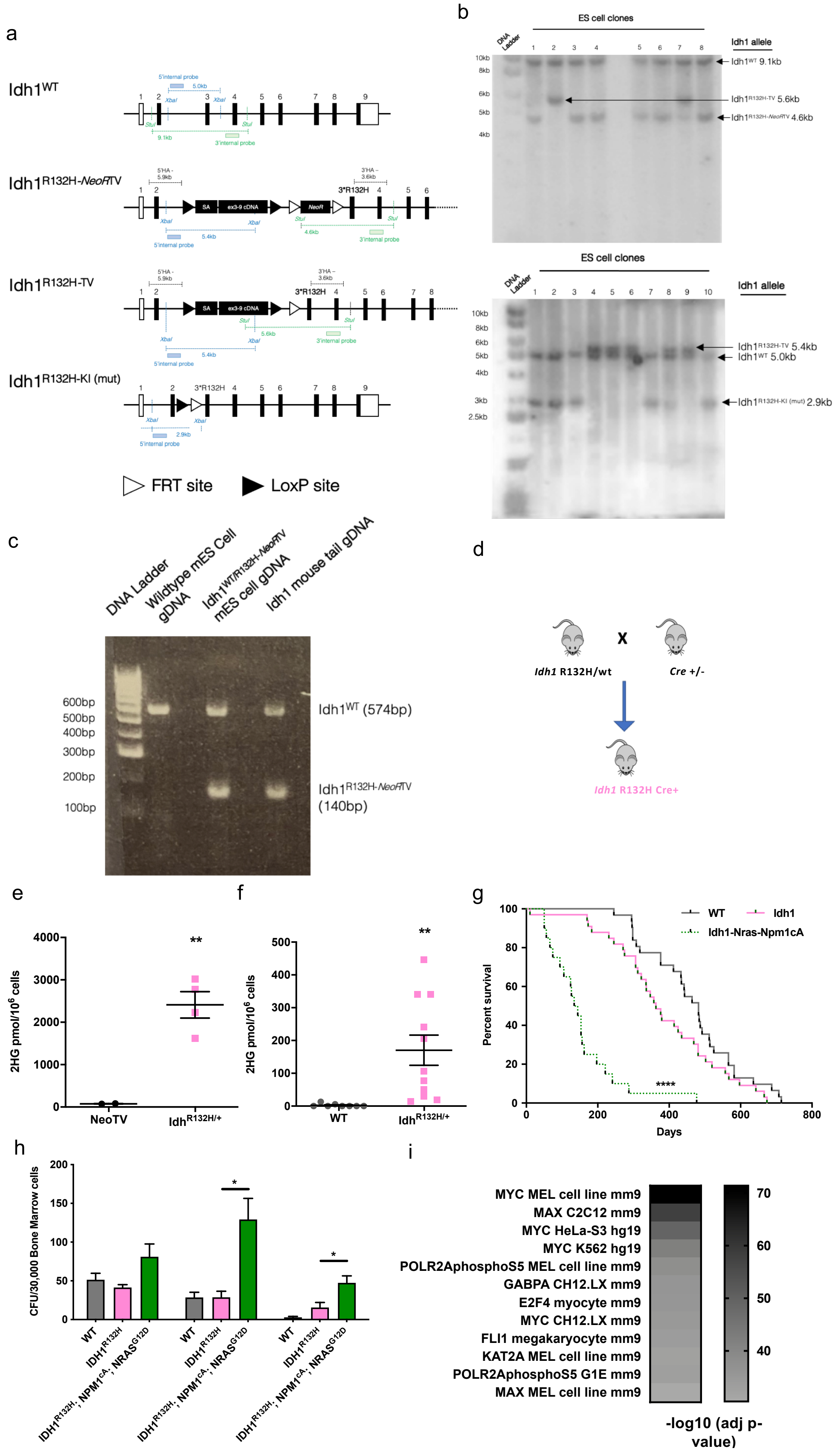
a
b
ES cell clones
Idh1 allele
c
DNA Ladder
Wildtype mES Cell gDNA
Idh1 mouse tail gDNA
Idh1WT (574bp)
d
Idh1 R132H/wt
Cre +/-
Idh1 R132H Cre+
e
f
g
h
i
FRT site
LoxP site
Percent survival
Days
WT
Idh1
Idh1-Nras-Npm1cA
MYC MEL cell line mm9
MAX C2C12 mm9
MYC HeLa-S3 hg19
MYC K562 hg19
POLR2AphosphoS5 MEL cell line mm9
GABPA CH12.LX mm9
E2F4 myocyte mm9
MYC CH12.LX mm9
FLI1 megakaryocyte mm9
KAT2A MEL cell line mm9
POLR2AphosphoS5 G1E mm9
MAX MEL cell line mm9
-log10 (adj p-value)

Fig. S1: Development and analysis of the *Idh1^{R132H}* pre-leukemia model. (A) Schematic of *Idh1^{WT}* and stepwise assembly of *Idh1^{R132H}* knock-in (KI) mutant (mut) allele, with representation of Southern Blotting probes used for confirmation of successful generation of intermediate *Idh1$^{R132H\text{-}NeoR\text{-}TV}$* and *Idh1$^{R132H\text{-}TV}$* alleles, as represented in panel B. **(B** Southern Blots confirming excision of the NeoR resistance cassette and LoxP recombination to generate *Idh1$^{R132H\text{-}NeoR\text{-}TV}$* and *Idh1$^{R132H\text{-}TV}$* alleles. **(C)** Genotyping agarose gel for selection of F1 mice carrying an inducible *Idh1$^{R132H\text{-}NeoR\text{-}TV}$* allele after backcross to *RosaFLPe*-negative mice, as described in Supplementary Methods. **(D)** Schematic of*Idh1^{R132H}* and *Mx1-Cre* mouse crosses to generate a *Cre* inducible *Idh1^{R132H}* mouse. **(E)** Mass spectrometry (MS) quantification of 2-Hydroxyglutarate (2-HG) in control (NeoTV) and *Idh1^{R132H}* (*Idh$^{R132H/+}$*) embryonic stem cells; mean ± SD, n=2-4 samples/condition. **(F)** MS quantification of 2-HG in control (WT) and *Idh1^{R132H}* (*Idh$^{R132H/+}$)* bone marrow (BM) cells; mean ± SD, n=8-11 animals/genotype, **p<0.01, 2-tailed t-test. **(G)** Survival curve of mice with *Idh1^{R132H}* or *Idh1^{R132H}-Nras^{G12D}-Npm1cA* alleles; n= 20-33 animals/genotype, ****p<0.0001, log-rank test. **(H)** Colony-forming assay of BM cells obtained from WT, *Idh1^{R132H}, Idh1R132H-Npm1^{cA}- Nras^{G12D}* animals, with serial re-plating; mean ± SD, n=4-10/genotype, *p<0.05, 2-tailed t-test. **(I)** EnrichR [47] analysis of Encode transcription factor binding enrichment in differentially down-regulated genes in *Idh1^{R132H}* leukemia vs pre-leukemia samples. RNA-seq analysis of *Idh1^{R132H}* and *Idh1R132H-Npm1^{cA}-NRAS^{G12D}* pre-leukemia (Lin$^-$ cells collected 4 weeks after pIpC treatment) vs. *Idh1R132H-Npm1^{cA}-NRAS^{G12D}* leukemia samples (terminal).

Fig S2

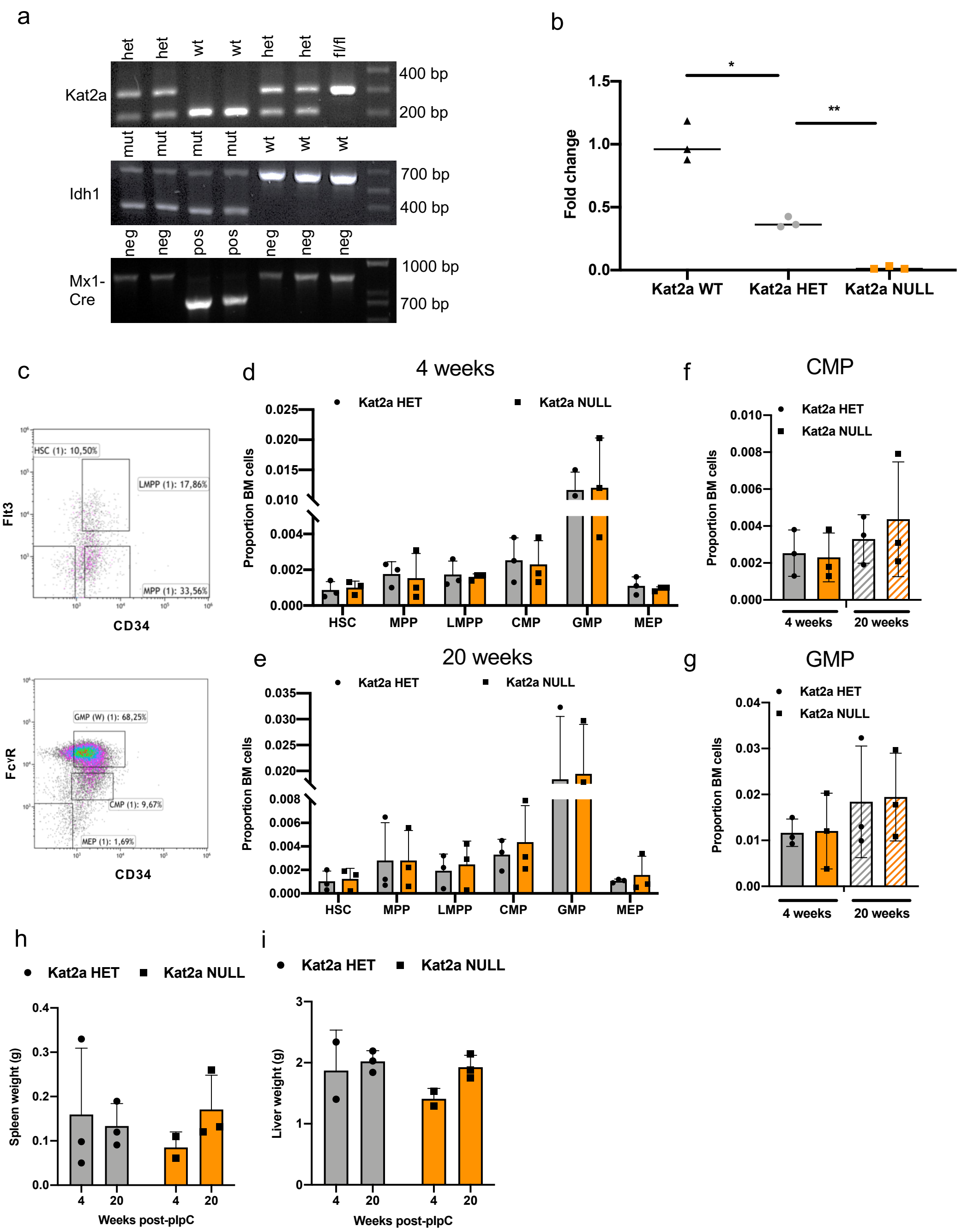

a
het het wt wt het het fl/fl
Kat2a
400 bp
200 bp
mut mut mut mut wt wt wt
Idh1
700 bp
400 bp
neg neg pos pos neg neg neg
Mx1-Cre
1000 bp
700 bp
b
Fold change
Kat2a WT
Kat2a HET
Kat2a NULL
c
Flt3
CD34
FcγR
d
4 weeks
Kat2a HET
Kat2a NULL
Proportion BM cells
HSC
MPP
LMPP
CMP
GMP
MEP
e
20 weeks
f
CMP
4 weeks
20 weeks
g
GMP
h
Spleen weight (g)
Weeks post-pIpC
i
Liver weight (g)

Fig. S2: Cellular characterization of *Idh1*^{R132H} pre-leukemia. (A) Representative PCR genotyping gel images of *Kat2a*, *Idh1* and *Mx1-Cre* alleles. **(B)** qRT-PCR analysis of *Kat2a* expression in BM cells of *Kat2aWT*, *Kat2aHET* and *Kat2aNULL* animals; mean ± SD, n=3 animals/genotype, *p<0.05, **p<0.01, 2-tailed t-test. **(C)** Flow cytometry gating strategy for enumeration of BM stem and progenitor cells in *Idh1* mut *Kat2a* HET and *Idh1* mut *Kat2a* NULL pre-leukemic animals. **(D)** Proportion of BM stem and progenitor cells in *Idh1* mut *Kat2a* HET and *Idh1* mut *Kat2a* NULL pre-leukemic animals 4 weeks post-pIpC treatment; mean ± SD, n=3, 2-tailed t-test. **(E)** Proportion of BM stem and progenitor cells in *Idh1* mut *Kat2a* HET and *Idh1* mut *Kat2a* NULL pre-leukemic animals 20-week post-pIpC treatment; mean ± SD, n=3, 2-tailed t-test. **(F)** Proportion of BM cells characterized as CMP population of cells at 4 week and 20-week time point; mean ± SD, n=3, 2-tailed t-test. **(G)** Proportion of BM cells characterized as GMP population of cells at 4 week and 20 week time point; mean ± SD, n=3, 2-tailed t-test. (**H-I)** Analysis of pre-leukemia burden 4 and 20-weeks post-pIpC, **(H)** spleen weights, **(I)** liver weights; mean ± SD, n=2-3.

Fig S3

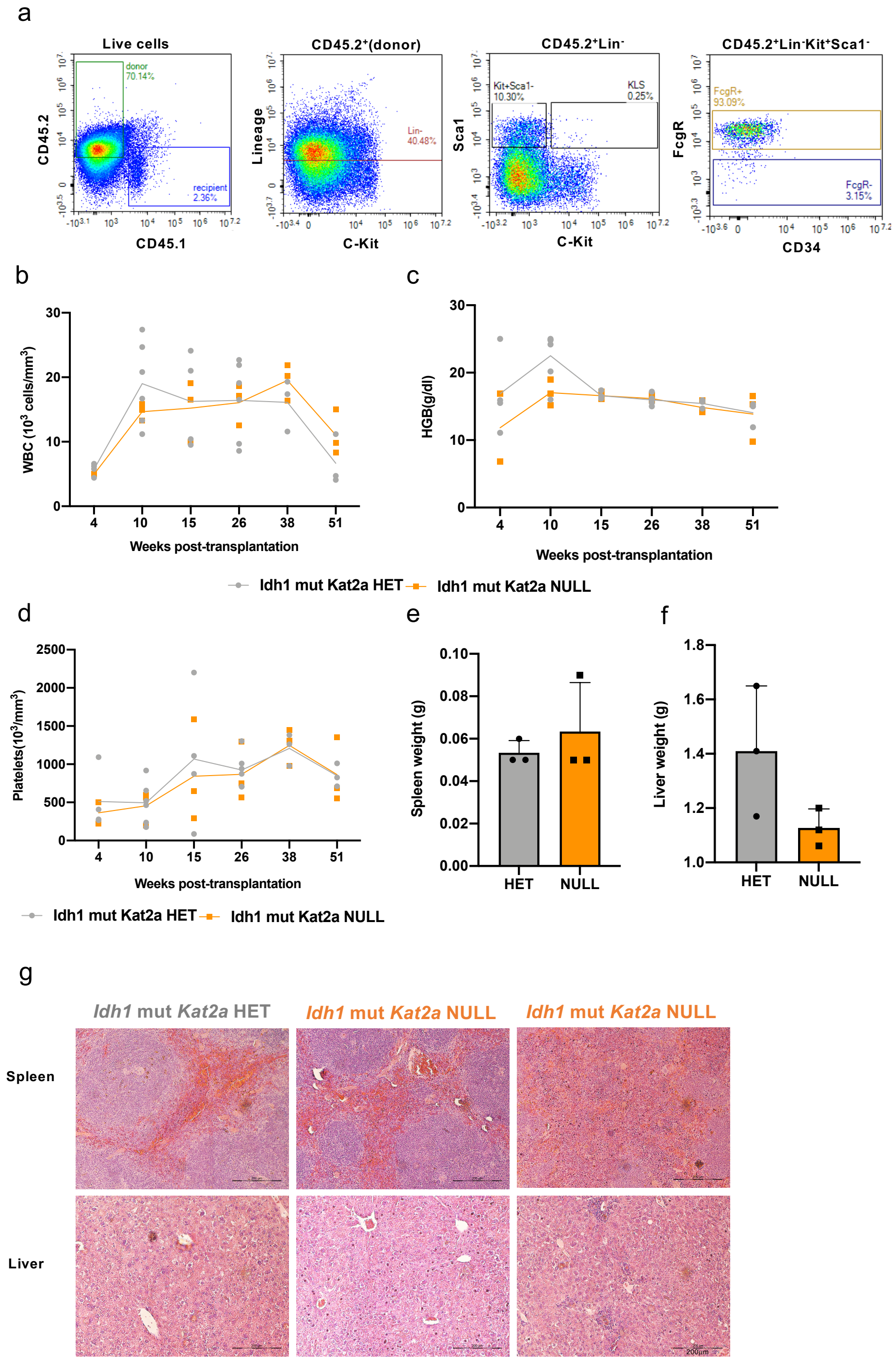
a
Live cells
CD45.2
CD45.1
donor
70.14%
recipient
2.36%
CD45.2⁺(donor)
Lineage
C-Kit
Lin-
40.48%
CD45.2⁺Lin⁻
Sca1
Kit+Sca1-
10.30%
KLS
0.25%
CD45.2⁺Lin⁻Kit⁺Sca1⁻
FcgR
CD34
FcgR+
93.09%
FcgR-
3.15%
b
WBC (10³ cells/mm³)
Weeks post-transplantation
c
HGB(g/dl)
Weeks post-transplantation
Idh1 mut Kat2a HET
Idh1 mut Kat2a NULL
d
Platelets(10³/mm³)
Weeks post-transplantation
Idh1 mut Kat2a HET
Idh1 mut Kat2a NULL
e
Spleen weight (g)
HET
NULL
f
Liver weight (g)
HET
NULL
g
Idh1 mut Kat2a HET
Idh1 mut Kat2a NULL
Idh1 mut Kat2a NULL
Spleen
Liver

Fig. S3: Investigation of *Idh1*^{R132H} pre-leukemia transplants. **(A)** Flow cytometry gating strategy for engrafted *Idh1* mut *Kat2a* HET and *Idh1* mut *Kat2a* NULL BM cells. **(B-D)** Analysis of hematological parameters from peripheral blood of mice engrafted with *Idh1* mut *Kat2a* HET and *Idh1* mut *Kat2a* NULL BM cells: **(B)** WBC, **(C)** Hemoglobin, **(D)** Platelets; mean ± SD, n=3-6 at 10 week time point, n.s. No animals (6 receiving *Idh1* mut *Kat2a* HET / 3 receiving *Idh1* mut *Kat2a* NULL cells) developed signs or symptoms or leukemia during the observation period. Three animals were culled for welfare reasons unrelated to a leukemia disease process. **(E-F)** Analysis of leukemia burden 1-year post-transplantation: **(E)** spleen weight, **(F)** liver weight; mean ± SD, n=3/genotype, 2-tailed t-test. **(G)** Histology analysis of *Idh1* mut *Kat2a* HET and *Idh1* mut *Kat2a* NULL recipient animals culled 1-year post-transplantation.

Fig S4

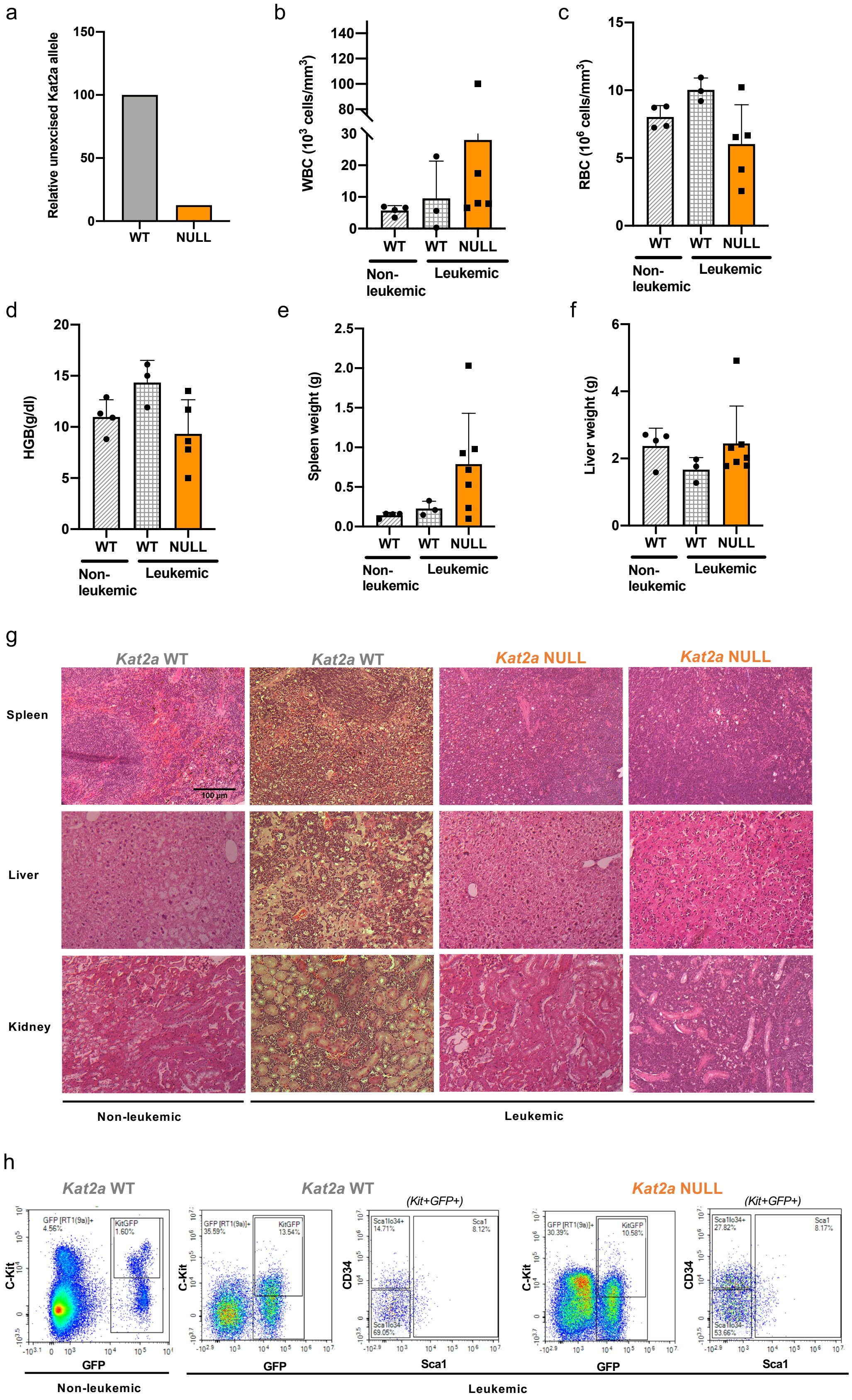

a
Relative unexcised Kat2a allele
WT
NULL
b
WBC (10^3 cells/mm^3)
c
RBC (10^6 cells/mm^3)
d
HGB(g/dl)
e
Spleen weight (g)
f
Liver weight (g)
WT
WT
NULL
Non-leukemic
Leukemic
g
Kat2a WT
Kat2a WT
Kat2a NULL
Kat2a NULL
Spleen
Liver
Kidney
100 μm
Non-leukemic
Leukemic
h
Kat2a WT
Kat2a WT
Kat2a NULL
(Kit+GFP+)
(Kit+GFP+)
C-Kit
CD34
GFP
Sca1
Non-leukemic
Leukemic

Fig. S4: Characterization of *RT1(9a) Kat2a* NULL leukemia. (A) Relative expression of *Kat2a* in *RT1(9a)*-transformed *Kat2aWT* and *Kat2aNULL* BM cells; transformation used pools of 3 animals/genotype. **(B-D)** Terminal peripheral blood analysis of mice engrafted with *RT1(9a)*-transduced *Kat2aWT* and *Kat2aNULL* Kit^+ / Lin^- cells and followed up for leukemia development. Animals were analysed upon exhibition of terminal symptoms, or after 405 days, if asymptomatic. **(B)** WBC, **(C)** RBC, **(D)** HGB; mean ± SD, n=3-5, 2-tailed t-test. **(E-F)** Analysis of leukemia burden at terminal time point in the same animals. **(E)** Spleen weight; **(F)** liver weight. **(G)** Representative histology images of spleen, liver, and kidney tissues of the same engrafted mouse cohort at terminal time point, with or without leukemia development. **(H)** Representative flow cytometry plots of terminal *Kat2aWT* or *Kat2aNULL RT1(9a)*-engrafted mice, with or without leukemia development. Leukemic RT1(9a) Kit^+ cells are predominantly $Sca1^-CD34^-$, as described [17].

Fig S5

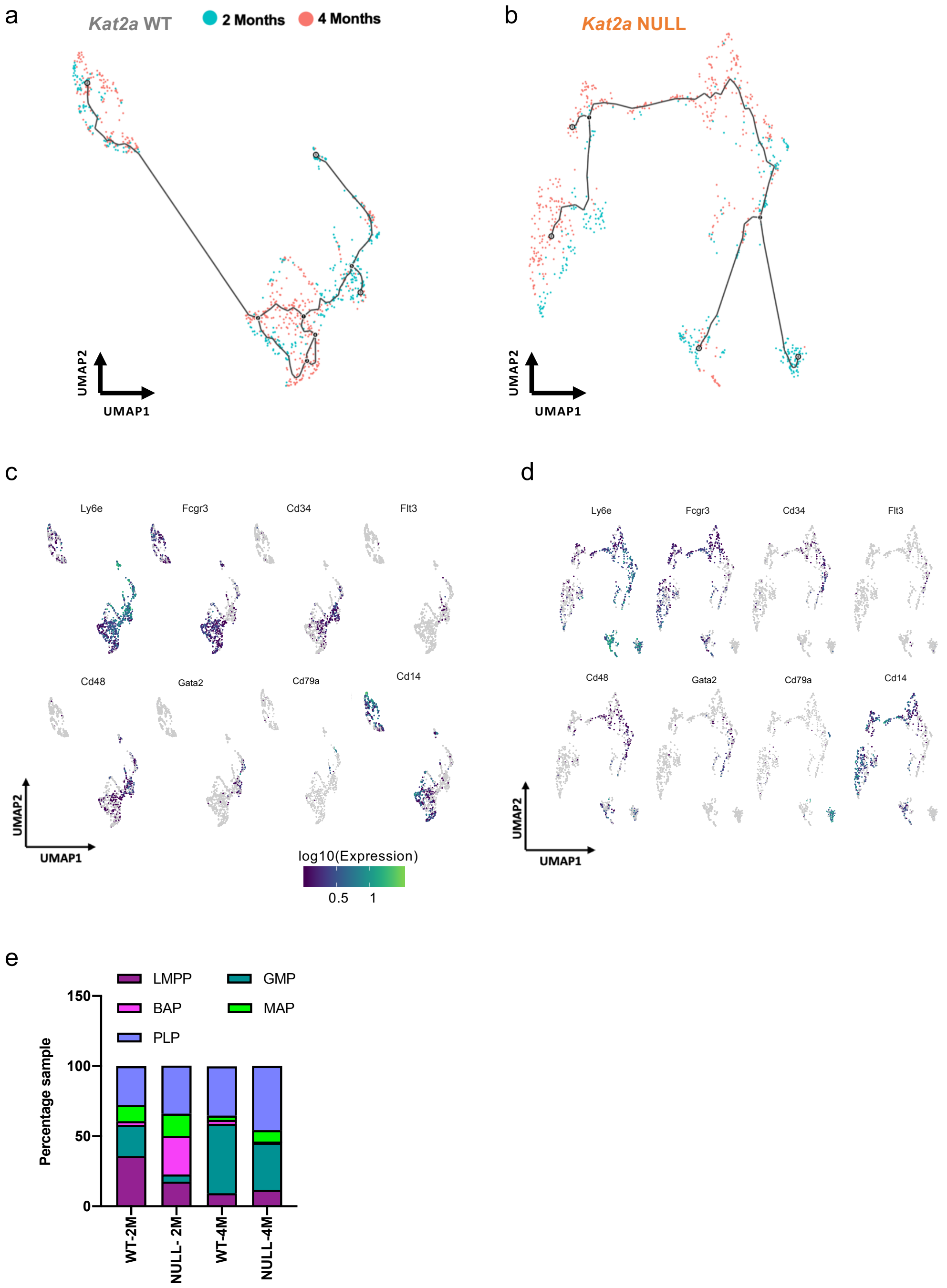
a
Kat2a WT
2 Months
4 Months
b
Kat2a NULL
UMAP2
UMAP1
c
Ly6e
Fcgr3
Cd34
Flt3
Cd48
Gata2
Cd79a
Cd14
log10(Expression)
0.5
1
d
e
LMPP
GMP
BAP
MAP
PLP
Percentage sample
150
100
50
0
WT-2M
NULL- 2M
WT-4M
NULL-4M

Fig. S5: Hematopoietic lineage-associated surface markers and cellular diversification along RT1(9a) pseudo-time trajectories. (A-B) Annotation of 2-month and 4-month samples along *Kat2a* WT (A) and *Kat2a* NULL pseudo-time trajectories. **(C-D)** Expression of lineage-affiliated hematopoietic markers in single-cell trajectories of RT1(9a) transduced BM; **(C)** *Kat2a* WT, **(D)** *Kat2a* NULL cells. *Ly6e*: hematopoietic stem cell (HSC), multipotent (MPP) and lympho/myeloid (LMPP) progenitors; myeloid cells). *Fcgr3*; myeloid progenitors, myelo-monocytic cells. *Cd34*: LMPP, MPP, myeloid progenitors, multipotent and lympho/myeloid progenitor marker), *Flt3*; MPP, LMPP, lymphoid cells. *Cd48*: committed progenitors and lymphocytes. *Cd14*; monocyte/macrophage. *Cd79a*: B lymphocytes. **(E)** Proportion of RT1(9a)-transduced cells in each individual time-point sample (*Kat2aWT* or *Kat2a*NULL, 2 or 4-months post-engraftment) contributed by candidate progenitor compartments captured by pseudo-time trajectory analysis. LMPP: lymphoid/myeloid-primed progenitor; GMP: granulocyte-monocyte progenitor; MAP: monocyte-affiliated progenitor; BAP: B-cell affiliated progenitor; PLP: Pre-leukemia progenitor.

Fig S6

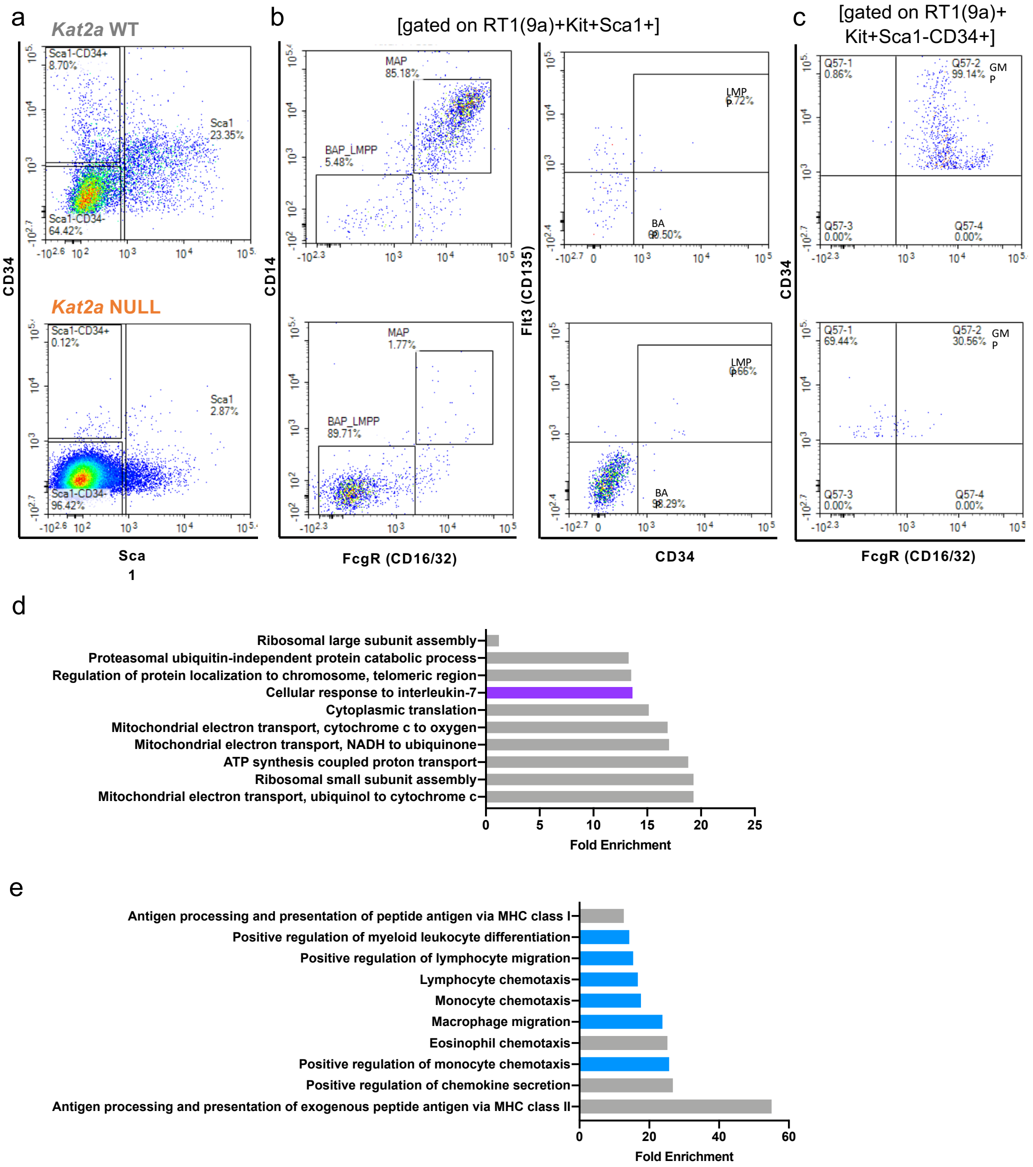

a
Kat2a WT
Sca1-CD34+
8.70%
Sca1
23.35%
Sca1-CD34-
64.42%
Kat2a NULL
Sca1-CD34+
0.12%
Sca1
2.87%
Sca1-CD34-
96.42%
CD34
Sca 1
b
[gated on RT1(9a)+Kit+Sca1+]
MAP
85.18%
BAP_LMPP
5.48%
MAP
1.77%
BAP_LMPP
89.71%
CD14
FcgR (CD16/32)
LMP
BA
98.29%
Flt3 (CD135)
CD34
c
[gated on RT1(9a)+ Kit+Sca1-CD34+]
Q57-1
0.86%
Q57-2
99.14%
GMP
Q57-3
0.00%
Q57-4
0.00%
Q57-1
69.44%
Q57-2
30.56%
Q57-3
0.00%
Q57-4
0.00%
CD34
FcgR (CD16/32)
d
Ribosomal large subunit assembly
Proteasomal ubiquitin-independent protein catabolic process
Regulation of protein localization to chromosome, telomeric region
Cellular response to interleukin-7
Cytoplasmic translation
Mitochondrial electron transport, cytochrome c to oxygen
Mitochondrial electron transport, NADH to ubiquinone
ATP synthesis coupled proton transport
Ribosomal small subunit assembly
Mitochondrial electron transport, ubiquinol to cytochrome c
0 5 10 15 20 25
Fold Enrichment
e
Antigen processing and presentation of peptide antigen via MHC class I
Positive regulation of myeloid leukocyte differentiation
Positive regulation of lymphocyte migration
Lymphocyte chemotaxis
Monocyte chemotaxis
Macrophage migration
Eosinophil chemotaxis
Positive regulation of monocyte chemotaxis
Positive regulation of chemokine secretion
Antigen processing and presentation of exogenous peptide antigen via MHC class II
0 20 40 60
Fold Enrichment

Fig. S6. Investigation of lymphoid and macrophage-affiliated compartments in RT1(9a) pre-leukemia and leukemia samples. (A-B) Flow cytometry analysis of terminal RT1(9a) mouse leukemias for LMPP, BAP and MAP cell surface markers as per Fig. S5C-D. Cells gated as C-kit$^+$ GFP [RT1(9a)]$^+$ (A) and C-kit$^+$ GFP [RT1(9a)]$^+$ Sca1$^{+/hi}$ (B), with right panel in (B) gated from BAP_LMPP. Samples are representative of *Kat2* WT and *Kat2a* NULL leukemias. **(C)** Flow cytometry analysis of terminal RT1(9a) mouse leukemias for GMP-like cell surface markers as per Fig. S5C-D. Cells gated as C-kit$^+$ GFP [RT1(9a)]$^+$ CD34$^+$Sca1$^{-/lo}$. Representative samples as in (A-B). **(D)** Gene ontology analysis of genes overexpressed in B-affiliated progenitors (BAP) using Panther14.0 [43]; *p-adj<0.05. **(E)** Gene ontology analysis of genes overexpressed in monocyte-affiliated progenitors (MAP) using Panther14.0 [43]; *p-adj<0.05.

Fig S7

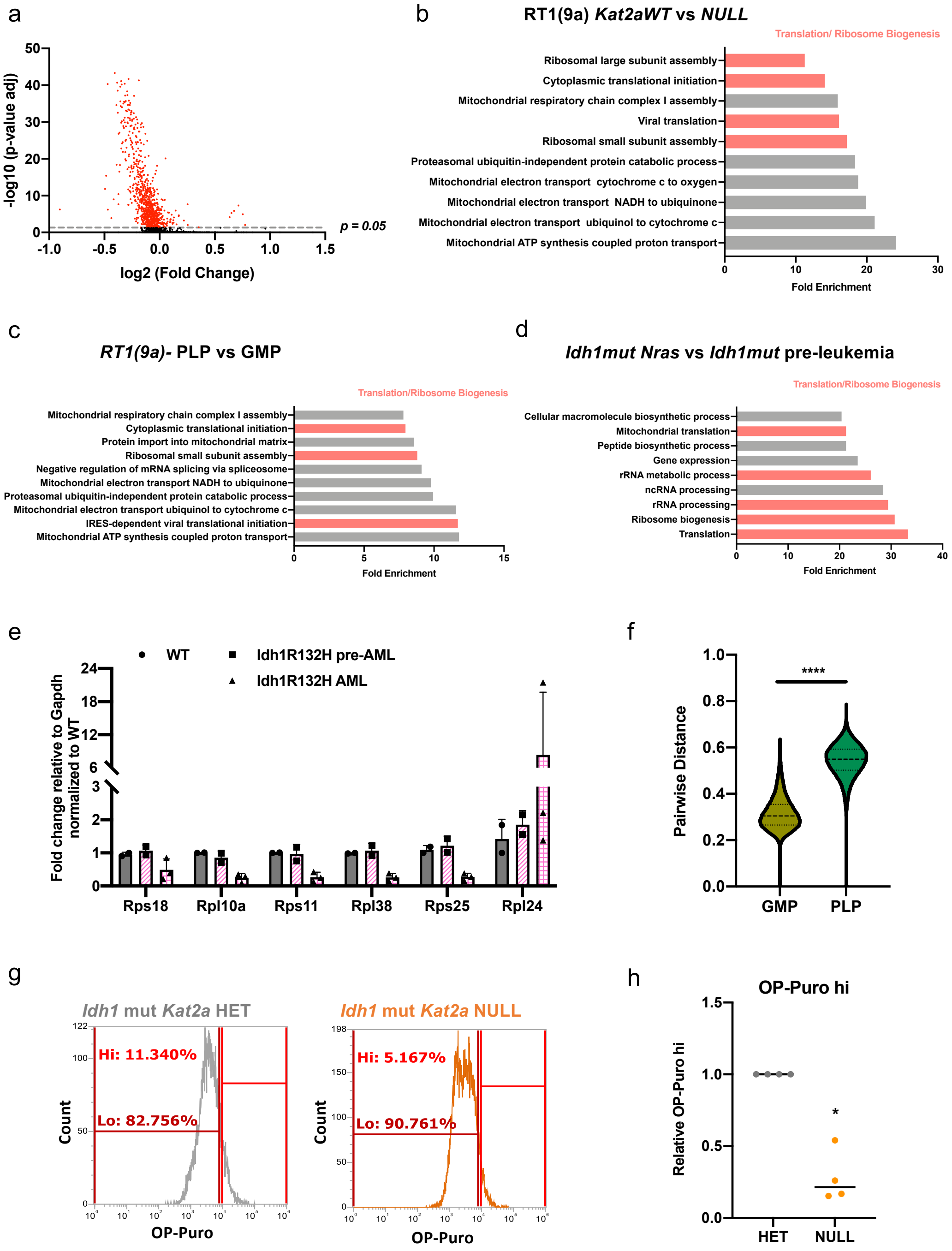

a
-log10 (p-value adj)
log2 (Fold Change)
p = 0.05
b
RT1(9a) Kat2aWT vs NULL
Translation/ Ribosome Biogenesis
Ribosomal large subunit assembly
Cytoplasmic translational initiation
Mitochondrial respiratory chain complex I assembly
Viral translation
Ribosomal small subunit assembly
Proteasomal ubiquitin-independent protein catabolic process
Mitochondrial electron transport cytochrome c to oxygen
Mitochondrial electron transport NADH to ubiquinone
Mitochondrial electron transport ubiquinol to cytochrome c
Mitochondrial ATP synthesis coupled proton transport
Fold Enrichment
c
RT1(9a)- PLP vs GMP
Translation/Ribosome Biogenesis
Mitochondrial respiratory chain complex I assembly
Cytoplasmic translational initiation
Protein import into mitochondrial matrix
Ribosomal small subunit assembly
Negative regulation of mRNA splicing via spliceosome
Mitochondrial electron transport NADH to ubiquinone
Proteasomal ubiquitin-independent protein catabolic process
Mitochondrial electron transport ubiquinol to cytochrome c
IRES-dependent viral translational initiation
Mitochondrial ATP synthesis coupled proton transport
Fold Enrichment
d
Idh1mut Nras vs Idh1mut pre-leukemia
Translation/Ribosome Biogenesis
Cellular macromolecule biosynthetic process
Mitochondrial translation
Peptide biosynthetic process
Gene expression
rRNA metabolic process
ncRNA processing
rRNA processing
Ribosome biogenesis
Translation
Fold Enrichment
e
WT
Idh1R132H pre-AML
Idh1R132H AML
Fold change relative to Gapdh normalized to WT
Rps18
Rpl10a
Rps11
Rpl38
Rps25
Rpl24
f
Pairwise Distance
****
GMP
PLP
g
Idh1 mut Kat2a HET
Hi: 11.340%
Lo: 82.756%
Count
OP-Puro
Idh1 mut Kat2a NULL
Hi: 5.167%
Lo: 90.761%
Count
OP-Puro
h
OP-Puro hi
Relative OP-Puro hi
*
HET
NULL

Fig. S7: *Kat2a* loss downregulates protein synthesis genes in pre-leukemia progression. (A) Volcano plot of single-cell RNA-seq data for RT1(9a) *Kat2aNULL* vs *WT* cells. Differentially expressed genes at p-adj <0.05 shown in red. **(B)** Over-represented gene ontologies (GO) for genes downregulated in RT1(9a) *Kat2aNULL* vs *WT* cells. Panther14.0 analysis of single-cell RNA-seq data, with 2 and 4-month timepoints analysed together; *p-adj<0.05. **(C)** Over-represented GO for genes down-regulated in RT1(9a) *Kat2aWT* PLP vs GMP *p-adj<0.05. **(D)** Over-represented GO for genes down-regulated in *N-Ras*^{G12D} *Idh1*^{R132H} and *Npm1c N-Ras*^{G12D} *Idh1*^{R132H} AML compared to *Idh1*^{R132H}, *Idh1*^{R132H} *Npm1c*, and *Npm1c N-Ras*^{G12D} *Idh1*^{R132H} pre-leukemia; *p-adj<0.05. Genes were not differential in *Idh1*^{R132H} vs *Idh1*^{WT}, or in *Idh1*^{R132H} *Npm1c* and *Npm1c N-Ras*^{G12D} *Idh1*^{R132H} vs *Idh1*^{R132H} pre-leukemia. **(E)** qRT-PCR analysis of ribosomal protein genes in *Idh1*^{R132H} leukemia vs. pre-leukemia samples; mean ± SD, n=2-3. Data from Lin^{-} BM plotted relative to *Idh1* WT Lin^{-} cells, normalised for *Gapdh* expression. **(F)** Comparison of pairwise distances between individual cells in GMP and PLP compartments using the ribosomal biogenesis signature in Fig. 6D-E; ****p<0.0001, non-parametric KS test. **(G)** Representative flow cytometry plots of OP-Puro incorporation in *Kat2aHET* vs. *Kat2aNULL Idh1*^{R132H} BM cells collected 20 weeks post-pIpC and treated *in vitro*. **(H)** Quantification of OP-Puro high cells in (G); data relative to *Kat2aHET Idh1*^{R132H}; mean ± SD, n=3, *p<0.05, 2-tailed t-test.

Supplementary Files 1-9 (Excel Spreadsheet – 9 tabs)

Supplementary File 1: List of genes post-filtration using Seurat

Supplementary File 2: List of genes utilized for pseudotime ordering analysis

Supplementary File 3: Differentially expressed genes in BAP cells compared to all other cells

Supplementary File 4: Differentially expressed genes in MAP cells compared to all other cells

Supplementary File 5: Differentially expressed genes in RT1(9a)-transformed *Kat2a* NULL cells relative to *Kat2a* WT

Supplementary File 6: Differentially expressed genes in RT1(9a)-transformed *Kat2a* NULL cells relative to *Kat2a* WT, within PLP cell compartment

Supplementary File 7: Differentially expressed genes in PLP compared to GMP

Supplementary File 8: Differentially expressed genes in $Nras^{G12D}$ $Idh1^{R132H}$ and *Npm1c* $Nras^{G12D}$ $Idh1^{R132H}$ AML compared to $Idh1^{R132H}$, $Idh1^{R132H}$ *Npm1c*, and *Npm1c* $Nras^{G12D}$ $Idh1^{R132H}$ pre-leukemia

Supplementary File 9: List of ribosomal biogenesis genes used for transcriptional variability and gene expression analysis in Fig. 5d and 5e.

REFERENCES AND NOTES

1. M. Greaves, C. C. Maley, Clonal evolution in cancer. *Nature* **481**, 306–313 (2012).

2. N. McGranahan, C. Swanton, Biological and therapeutic impact of intratumor heterogeneity in cancer evolution. *Cancer Cell* **27**, 15–26 (2015).

3. N. Zahir, R. Sun, D. Gallahan, R. A. Gatenby, C. Curtis, Characterizing the ecological and evolutionary dynamics of cancer. *Nat. Genet.* **52**, 759–767 (2020).

4. E. Persi, Y. I. Wolf, D. Horn, E. Ruppin, F. Demichelis, R. A. Gatenby, R. J. Gillies, E. V. Koonin, Mutation–Selection balance and compensatory mechanisms in tumour evolution. *Nat. Rev. Genet.* **22**, 251–262 (2020).

5. D. A. Landau, K. Clement, M. J. Ziller, P. Boyle, J. Fan, H. Gu, K. Stevenson, C. Sougnez, L. Wang, S. Li, D. Kotliar, W. Zhang, M. Ghandi, L. Garraway, S. M. Fernandes, K. J. Livak, S. Gabriel, A. Gnirke, E. S. Lander, J. R. Brown, D. Neuberg, P. V. Kharchenko, N. Hacohen, G. Getz, A. Meissner, C. J. Wu, Locally disordered methylation forms the basis of intratumor methylome variation in chronic lymphocytic leukemia. *Cancer Cell* **26**, 813–825 (2014).

6. H. Pan, Y. Jiang, M. Boi, F. Tabbò, D. Redmond, K. Nie, M. Ladetto, A. Chiappella, L. Cerchietti, R. Shaknovich, A. M. Melnick, G. G. Inghirami, W. Tam, O. Elemento, Epigenomic evolution in diffuse large B-cell lymphomas. *Nat. Commun.* **6**, 6921 (2015).

7. Cancer Genome Atlas Research Network, T. J. Ley, C. Miller, L. Ding, B. J. Raphael, A. J. Mungall, A. G. Robertson, K. Hoadley, T. J. Triche Jr., P. W. Laird, J. D. Baty, L. L. Fulton, R. Fulton, S. E. Heath, J. Kalicki-Veizer, C. Kandoth, J. M. Klco, D. C. Koboldt, K.-L. Kanchi, S. Kulkarni, T. L. Lamprecht, D. E. Larson, L. Lin, C. Lu, M. D. McLellan, J. F. McMichael, J. Payton, H. Schmidt, D. H. Spencer, M. H. Tomasson, J. W. Wallis, L. D. Wartman, M. A. Watson, J. Welch, M. C. Wendl, A. Ally, M. Balasundaram, I. Birol, Y. Butterfield, R. Chiu, A. Chu, E. Chuah, H.-J. Chun, R. Corbett, N. Dhalla, R. Guin, A. He, C. Hirst, M. Hirst, R. A. Holt, S. Jones, A. Karsan, D. Lee, H. I. Li, M. A. Marra, M. Mayo, R. A. Moore, K. Mungall, J. Parker, E. Pleasance, P. Plettner, J. Schein, D. Stoll, L. Swanson, A. Tam, N. Thiessen, R. Varhol, N. Wye, Y. Zhao, S. Gabriel, G. Getz, C. Sougnez, L. Zou, M. D. M. Leiserson, F. Vandin, H.-T. Wu, F. Applebaum, S. B. Baylin, R. Akbani,

B. M. Broom, K. Chen, T. C. Motter, K. Nguyen, J. N. Weinstein, N. Zhang, M. L. Ferguson, C. Adams, A. Black, J. Bowen, J. Gastier-Foster, T. Grossman, T. Lichtenberg, L. Wise, T. Davidsen, J. A. Demchok, K. R. M. Shaw, M. Sheth, H. J. Sofia, L. Yang, J. R. Downing, G. Eley, Genomic and epigenomic landscapes of adult de novo acute myeloid leukemia. *N. Engl. J. Med.* **368**, 2059–2074 (2013).

8. M. A. Dawson, R. K. Prinjha, A. Dittmann, G. Giotopoulos, M. Bantscheff, W.-I. Chan, S. C. Robson, C.-w. Chung, C. Hopf, M. M. Savitski, C. Huthmacher, E. Gudgin, D. Lugo, S. Beinke, T. D. Chapman, E. J. Roberts, P. E. Soden, K. R. Auger, O. Mirguet, K. Doehner, R. Delwel, A. K. Burnett, P. Jeffrey, G. Drewes, K. Lee, B. J. P. Huntly, T. Kouzarides, Inhibition of BET recruitment to chromatin as an effective treatment for MLL-fusion leukaemia. *Nature* **478**, 529–533 (2011).

9. K. M. Bernt, N. Zhu, A. U. Sinha, S. Vempati, J. Faber, A. V. Krivtsov, Z. Feng, N. Punt, A. Daigle, L. Bullinger, R. M. Pollock, V. M. Richon, A. L. Kung, S. A. Armstrong, MLL-rearranged leukemia is dependent on aberrant H3K79 methylation by DOT1L. *Cancer Cell* **20**, 66–78 (2011).

10. L. I. Shlush, S. Zandi, A. Mitchell, W. C. Chen, J. M. Brandwein, V. Gupta, J. A. Kennedy, A. D. Schimmer, A. C. Schuh, K. W. Yee, J. L. McLeod, M. Doedens, J. J. F. Medeiros, R. Marke, H. J. Kim, K. Lee, J. D. McPherson, T. J. Hudson; HALT Pan-Leukemia Gene Panel Consortium, A. M. K. Brown, F. Yousif, Q. M. Trinh, L. D. Stein, M. D. Minden, J. C. Y. Wang, J. E. Dick, Identification of pre-leukaemic haematopoietic stem cells in acute leukaemia. *Nature* **506**, 328–333 (2014).

11. M. R. C.-Zimmerman, W.-J. Hong, I. L. Weissman, B. C. Medeiros, R. Majeti, Preleukemic mutations in human acute myeloid leukemia affect epigenetic regulators and persist in remission. *Proc. Natl. Acad. Sci. U.S.A.* **111**, 2548–2553 (2014).

12. A. F. Domingues, R. Kulkarni, G. Giotopoulos, S. Gupta, L. Vinnenberg, L. Arede, E. Foerner, M. Khalili, R. R. Adao, A. Johns, S. Tan, K. Zeka, B. J. Huntly, S. Prabakaran, C. Pina, Loss of KAT2A enhances transcriptional noise and depletes acute myeloid leukemia stem-like cells. *eLife* **9**, e51754 (2020).

13. K. Tzelepis, H. Koike-Yusa, E. De Braekeleer, Y. Li, E. Metzakopian, O. M. Dovey, A. Mupo, V. Grinkevich, M. Li, M. Mazan, M. Gozdecka, S. Ohnishi, J. Cooper, M. Patel, T. M. Kerrell, B. Chen, A. F. Domingues, P. Gallipoli, S. Teichmann, H. Ponstingl, U. M. Dermott, J. Saez-Rodriguez, B. J. P. Huntly, F. Iorio, C. Pina, G. S. Vassiliou, K. Yusa, A CRISPR dropout screen identifies genetic vulnerabilities and therapeutic targets in acute myeloid leukemia. *Cell Rep.* **17**, 1193–1205 (2016).

14. N. Moris, S. Edri, D. Seyres, R. Kulkarni, A. F. Domingues, T. Balayo, M. Frontini, C. Pina, Histone acetyltransferase KAT2A stabilizes pluripotency with control of transcriptional heterogeneity. *Stem Cells* **36**, 1828–1838 (2018).

15. T. C. P. Somervaille, M. L. Cleary. Identification and characterization of leukemia stem cells in murine MLL-AF9 acute myeloid leukemia. *Cancer Cell* **10**, 257–268 (2006).

16. E. Papaemmanuil, M. Gerstung, L. Bullinger, V. I. Gaidzik, P. Paschka, N. D. Roberts, N. E. Potter, M. Heuser, F. Thol, N. Bolli, G. Gundem, P. Van Loo, I. Martincorena, P. Ganly, L. Mudie, S. M. Laren, S. O.'Meara, K. Raine, D. R. Jones, J. W. Teague, A. P. Butler, M. F. Greaves, A. Ganser, K. Döhner, R. F. Schlenk, H. Döhner, P. J. Campbell, Genomic classification and prognosis in acute myeloid leukemia. *N. Engl. J. Med.* **374**, 2209–2221 (2016).

17. M. Yan, E. Kanbe, L. F. Peterson, A. Boyapati, Y. Miao, Y. Wang, I.-M. Chen, Z. Chen, J. D. Rowley, C. L. Willman, D.-E. Zhang, A previously unidentified alternatively spliced isoform of t(8;21) transcript promotes leukemogenesis. *Nat. Med.* **12**, 945–949 (2006).

18. P. Paschka, R. F. Schlenk, V. I. Gaidzik, M. Habdank, J. Krönke, L. Bullinger, D. Späth, S. Kayser, M. Zucknick, K. Götze, H.-A. Horst, U. Germing, H. Döhner, K. Döhner, IDH1 and IDH2 mutations are frequent genetic alterations in acute myeloid leukemia and confer adverse prognosis in cytogenetically normal acute myeloid leukemia with NPM1 mutation without FLT3 internal tandem duplication. *J. Clin. Oncol.* **28**, 3636–3643 (2010).

19. K. D. Rasmussen, G. Jia, J. V. Johansen, M. T. Pedersen, N. Rapin, F. O. Bagger, B. T. Porse, O. A. Bernard, J. Christensen, K. Helin, Loss of TET2 in hematopoietic cells leads to DNA

hypermethylation of active enhancers and induction of leukemogenesis. *Genes Dev.* **29**, 910–922 (2015).

20. F. Basheer, G. Giotopoulos, E. Meduri, H. Yun, M. Mazan, D. Sasca, P. Gallipoli, L. Marando, M. Gozdecka, R. Asby, O. Sheppard, M. Dudek, L. Bullinger, H. Döhner, R. Dillon, S. Freeman, O. Ottmann, A. Burnett, N. Russell, E. Papaemmanuil, R. Hills, P. Campbell, G. S. Vassiliou, B. J. P. Huntly, Contrasting requirements during disease evolution identify EZH2 as a therapeutic target in AML. *J. Exp. Med.* **216**, 966–981 (2019).

21. C. Trapnell, D. Cacchiarelli, J. Grimsby, P. Pokharel, S. Li, M. Morse, N. J. Lennon, K. J. Livak, T. S. Mikkelsen, J. L. Rinn, The dynamics and regulators of cell fate decisions are revealed by pseudotemporal ordering of single cells. *Nat. Biotechnol.* **32**, 381–386 (2014).

22. J. Adolfsson, R. Månsson, N. Buza-Vidas, A. Hultquist, K. Liuba, C. T. Jensen, D. Bryder, L. Yang, O.-J. Borge, L. A. M. Thoren, K. Anderson, E. Sitnicka, Y. Sasaki, M. Sigvardsson, S. Eirik, W. Jacobsen, Identification of $Flt3^+$ lympho-myeloid stem cells lacking erythro-megakaryocytic potential a revised road map for adult blood lineage commitment. *Cell* **121**, 295–306 (2005).

23. K. Akashi, D. Traver, T. Miyamoto, I. L. Weissman, A clonogenic common myeloid progenitor that gives rise to all myeloid lineages. *Nature* **404**, 193–197 (2000).

24. A. Ptasinska, S. A. Assi, D. Mannari, S. R. James, D. Williamson, J. Dunne, M. Hoogenkamp, M. Wu, M. Care, H. McNeill, P. Cauchy, M. Cullen, R. M. Tooze, D. G. Tenen, B. D. Young, P. N. Cockerill, D. R. Westhead, O. Heidenreich, C. Bonifer, Depletion of RUNX1/ETO in t(8;21) AML cells leads to genome-wide changes in chromatin structure and transcription factor binding. *Leukemia* **26**, 1829–1841 (2012).

25. G. L. Manno, R. Soldatov, A. Zeisel, E. Braun, H. Hochgerner, V. Petukhov, K. Lidschreiber, M. E. Kastriti, P. Lönnerberg, A. Furlan, J. Fan, L. E. Borm, Z. Liu, D. van Bruggen, J. Guo, X. He, R. Barker, E. Sundström, G. Castelo-Branco, P. Cramer, I. Adameyko, S. Linnarsson, P. V. Kharchenko, RNA velocity of single cells. *Nature* **560**, 494–498 (2018).

26. J. M. Raser, E. K. O'Shea, Control of stochasticity in eukaryotic gene expression. *Science* **304**, 1811–1814 (2004).

27. H. Mohammed, I. Hernando-Herraez, A. Savino, J. Nichols, J. C. Marioni, W. Reik, Single-cell landscape of transcriptional heterogeneity and cell fate decisions during mouse early gastrulation. *Cell Rep.* **20**, 1215–1228 (2017).

28. A. A. Kolodziejczyk, J. K. Kim, J. C. H. Tsang, T. Ilicic, J. Henriksson, K. N. Natarajan, A. C. Tuck, X. Gao, M. Bühler, P. Liu, J. C. Marioni, S. A. Teichmann, Single cell RNA-sequencing of pluripotent states unlocks modular transcriptional variation. *Cell Stem Cell* **17**, 471–485 (2015).

29. X. Cai, L. Gao, L. Teng, J. Ge, Z. M. Oo, A. R. Kumar, D. G. Gilliland, P. J. Mason, K. Tan, N. A. Speck, Runx1 deficiency decreases ribosome biogenesis and confers stress resistance to hematopoietic stem and progenitor cells. *Cell Stem Cell* **17**, 165–177 (2015).

30. F. Christen, K. Hoyer, K. Yoshida, H.-A. Hou, N. Waldhueter, M. Heuser, R. K. Hills, W. Chan, R. Hablesreiter, O. Blau, Y. Ochi, P. Klement, W.-C. Chou, I.-W. Blau, J.-L. Tang, T. Zemojtel, Y. Shiraishi, Y. Shiozawa, F. Thol, A. Ganser, B. Löwenberg, D. C. Linch, L. Bullinger, P. J. M. Valk, H.-F. Tien, R. E. Gale, S. Ogawa, F. Damm, Genomic landscape and clonal evolution of acute myeloid leukemia with t(8;21): An international study on 331 patients. *Blood* **133**, 1140–1151 (2019).

31. A. Mandoli, A. A. Singh, K. H. M. Prange, E. Tijchon, M. Oerlemans, R. Dirks, M. T. Huurne, A. T. J. Wierenga, E. M. Jannsen-Megens, K. Berentsen, N. Sharifi, B. Kim, F. Matarese, L. N. Nguyen, N. C. Hubner, N. A. Rao, E. van den Akker, L. Altucci, E. Vellenga, H. G. Stunnenberg, J. H. A. Martens, The hematopoietic transcription factors RUNX1 and ERG prevent AML1-ETO oncogene overexpression and onset of the apoptosis program in t(8;21) AMLs. *Cell Rep.* **17**, 2087–2100 (2016).

32. C. Wichmann, I. Q.-L. Coco, Ö. Yildiz, L. Chen-Wichmann, H. Weber, T. Syzonenko, C. Döring, C. Brendel, K. Ponnusamy, A. Kinner, C. Brandts, R. Henschler, M. Grez, Activating c-KIT mutations confer oncogenic cooperativity and rescue RUNX1/ETO-induced DNA damage and apoptosis in human primary $CD34^+$ hematopoietic progenitors. *Leukemia* **29**, 279–289 (2015).

33. L. Arede, E. Foerner, S. Wind, R. Kulkarni, A. F. Domingues, G. Giotopoulos, S. Kleinwaechter, M. Mollenhauer-Starkl, H. Davison, A. Chandru, R. Asby, R. Samarista, S. Gupta, D. Forte, A. Curti, E. Scheer, B. J. P. Huntly, L. Tora, C. Pina, KAT2A complexes ATAC and SAGA play unique roles in cell maintenance and identity in hematopoiesis and leukemia. *Blood Adv.* **6**, 165–180 (2022).

34. S. Ecker, V. Pancaldi, D. Rico, A. Valencia, Higher gene expression variability in the more aggressive subtype of chronic lymphocytic leukemia. *Genome Med.* **7**, 8 (2015).

35. S. Gachet, T. el-Chaar, D. Avran, E. Genesca, F. Catez, S. Quentin, M. Delord, G. Thérizols, D. Briot, G. Meunier, L. Hernandez, M. Pla, W. K. Smits, J. G. Buijs-Gladdines, W. van Loocke, G. Menschaert, I. André-Schmutz, T. Taghon, P. van Vlierberghe, J. P. Meijerink, A. Baruchel, H. Dombret, E. Clappier, J. J. Diaz, C. Gazin, H. de Thé, F. Sigaux, J. Soulier, Deletion 6q drives T-cell leukemia progression by ribosome modulation. *Cancer Discov.* **8**, 1614–1631 (2018).

36. R. A. J. Signer, J. A. Magee, A. Salic, S. J. Morrison, Haematopoietic stem cells require a highly regulated protein synthesis rate. *Nature* **508**, 49–54 (2014).

37. C. P. Mill, W. Fiskus, C. D. DiNardo, C. Birdwell, J. A. Davis, T. M. Kadia, K. Takahashi, N. Short, N. Daver, M. Ohanian, G. Borthakur, S. M. Kornblau, M. R. Green, Y. Qi, X. Su, J. D. Khoury, K. N. Bhalla, Effective therapy for AML with RUNX1 mutation by cotreatment with inhibitors of protein translation and BCL2. *Blood* **139**, 907–921 (2022).

38. W. C. Skarnes, B. Rosen, A. P. West, M. Koutsourakis, W. Bushell, V. Iyer, A. O. Mujica, M. Thomas, J. Harrow, T. Cox, D. Jackson, J. Severin, P. Biggs, J. Fu, M. Nefedov, P. J. de Jong, A. F. Stewart, A. Bradley, A conditional knockout resource for the genome-wide study of mouse gene function. *Nature* **474**, 337–342 (2011).

39. C. I. Rodríguez, F. Buchholz, J. Galloway, R. Sequerra, J. Kasper, R. Ayala, A. F. Stewart, S. M. Dymecki, High-efficiency deleter mice show that FLPe is an alternative to Cre-loxP. *Nat. Genet.* **25**, 139–140 (2000).

40. O. M. Dovey, J. L. Cooper, A. Mupo, C. S. Grove, C. Lynn, N. Conte, R. M. Andrews, S. Pacharne, K. Tzelepis, M. S. Vijayabaskar, P. Green, R. Rad, M. Arends, P. Wright, K. Yusa, A. Bradley, I.

Varela, G. S. Vassiliou, Molecular synergy underlies the co-occurrence patterns and phenotype of NPM1-mutant acute myeloid leukemia. *Blood* **130**, 1911–1922 (2017).

41. A. Butler, P. Hoffman, P. Smibert, E. Papalexi, R. Satija, Integrating single-cell transcriptomic data across different conditions, technologies, and species. *Nat. Biotechnol.* **36**, 411–420 (2018).

42. M. I. Love, W. Huber, S. Anders, Moderated estimation of fold change and dispersion for RNA-seq data with DESeq2. *Genome Biol.* **15**, 550 (2014).

43. H. Mi, A. Muruganujan, D. Ebert, X. Huang, P. D. Thomas, PANTHER version 14: More genomes, a new PANTHER GO-slim and improvements in enrichment analysis tools. *Nucleic Acids Res.* **47**, D419–D426 (2019).

44. A. Dobin, C. A. Davis, F. Schlesinger, J. Drenkow, C. Zaleski, S. Jha, P. Batut, M. Chaisson, T. R. Gingeras, STAR: Ultrafast universal RNA-seq aligner. *Bioinformatics* **29**, 15–21 (2013).

45. J. Harrow, A. Frankish, J. M. Gonzalez, E. Tapanari, M. Diekhans, F. Kokocinski, B. L. Aken, D. Barrell, A. Zadissa, S. Searle, I. Barnes, A. Bignell, V. Boychenko, T. Hunt, M. Kay, G. Mukherjee, J. Rajan, G. Despacio-Reyes, G. Saunders, C. Steward, R. Harte, M. Lin, C. Howald, A. Tanzer, T. Derrien, J. Chrast, N. Walters, S. Balasubramanian, B. Pei, M. Tress, J. M. Rodriguez, I. Ezkurdia, J. van Baren, M. Brent, D. Haussler, M. Kellis, A. Valencia, A. Reymond, M. Gerstein, R. Guigó, T. J. Hubbard, GENCODE: The reference human genome annotation for the ENCODE project. *Genome Res.* **22**, 1760–1774 (2012).

46. S. Anders, P. T. Pyl, W. Huber, HTSeq--A Python framework to work with high-throughput sequencing data. *Bioinformatics* **31**, 166–169 (2015).

47. Z. Xie, A. Bailey, M. V. Kuleshov, D. J. B. Clarke, J. E. Evangelista, S. L. Jenkins, A. Lachmann, M. L. Wojciechowicz, E. Kropiwnicki, K. M. Jagodnik, M. Jeon, A. Ma’ayan, Gene set knowledge discovery with enrichr. *Curr. Protoc.* **1**,e90 (2021).
